# From waste rubber to value polybutadiene modification for circular materials

**DOI:** 10.1038/s41467-026-69032-9

**Published:** 2026-02-05

**Authors:** Janna Jeschke, Hatice Mutlu

**Affiliations:** 1 Institut de Science des Matériaux de Mulhouse (IS2M), UMR 7361, Université de Haute Alsace (UHA), Mulhouse, France; 2 Technical Polymer Chemistry, Department of Chemistry, Rheinland-Pfälzische Technische Universität Kaiserslautern-Landau (RPTU), Kaiserslautern, Germany; 3 Leibniz-Institut für Verbundwerkstoffe (IVW) GmbH, Kaiserslautern, Germany

**Keywords:** Polymer synthesis, Polymers

## Abstract

Synthetic rubber waste poses a growing environmental challenge due to its cross-linked, non-recyclable nature. This Perspective examines emerging post-polymerization modification strategies for linear polybutadiene and vulcanized rubber, spanning catalytic, metal-free, and catalyst-free approaches, and evaluates their alignment with Green and Circular Chemistry principles. By coupling sustainability metrics with assessments of technological readiness, we highlight current limitations and opportunities for the real-world implementation of these approaches. Looking ahead, we outline how integrating mechanochemistry, machine learning, and life-cycle assessment can enable scalable, low-impact transformations that recast vulcanized polybutadiene from waste to resource, paving the way toward a circular elastomer framework.

## Abstract (web summary)

Synthetic rubber poses a growing environmental challenge due to its non-recyclable nature. This Perspective examines emerging post-polymerization modification strategies for rubber waste spanning catalytic, metal-free, and catalyst-free approaches, and evaluates their alignment with Green and Circular Chemistry principles.

## Introduction

**Fig. 1:**
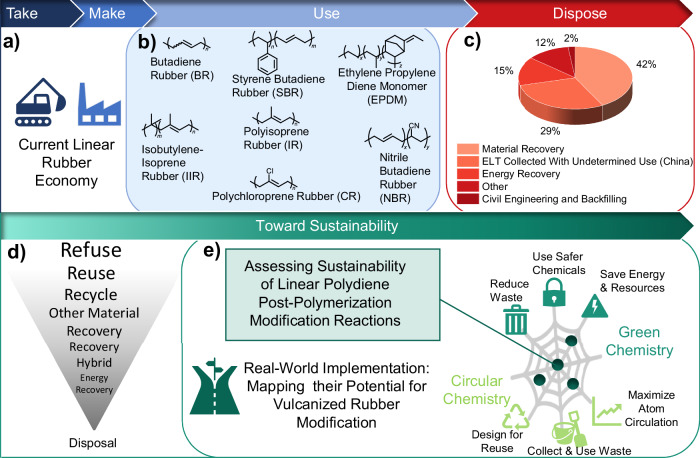
Current linear rubber economy, characterized by a “take-make-use-dispose” model and potential routes to guide the development and implementation of sustainable vulcanized rubber valorization methods. **a** Derived from petroleum-based feedstocks, monomers such as 1,3-butadiene are polymerized to yield linear polydienes, which are used as a building block, e.g., to undergo vulcanization, and to build up cross-linked materials. **b** Chemical structures of relevant commercial polydiene homo- and copolymers, covering a wide range of applications (e.g., vulcanized styrene butadiene rubber (SBR) in car tires). **c** Current waste management of end-of-life tires (ELTs)^20^. **d** Waste hierarchy to guide the transition towards a circular rubber economy. **e** Based on the frameworks of Green and Circular Chemistry, recent linear and vulcanized polydiene post-polymerization modifications are evaluated in this perspective, focusing on their potential to address the chemical modification of post-consumer vulcanized rubber waste.

**Fig. 2:**
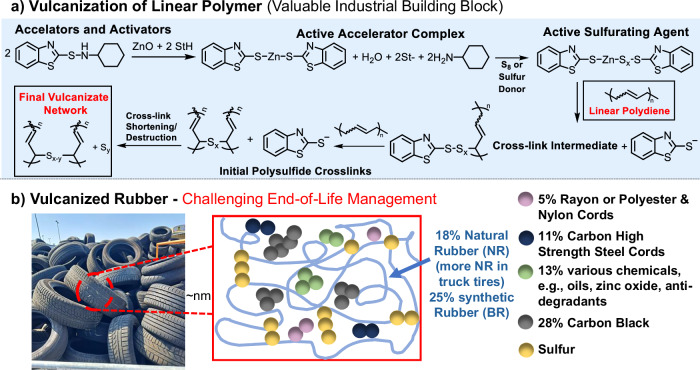
Vulcanization of linear polydienes and material composition of a tire. **a** According to Morrison and Porter^11^, vulcanization proceeds via the following steps: (1) formation of an active accelerator complex through the interaction of zinc oxide (ZnO), the co-activator stearic acid (StH), and the accelerator *N*-Cyclohexyl-2-benzothiazole sulfenamide (CBS); (2) reaction of the aforementioned complex with sulfur to generate an active sulfurating species; (3) yielding cross-link intermediates through the interaction between the sulfurating agent and linear butadiene rubber. (4) formation of polysulfide cross-links, which gradually transform into shorter, more stable sulfur bridges. **b** Material composition of tires, consisting of vulcanized rubber and diverse additives (such as rayon and nylon cords, zinc oxide, carbon black, and sulfur amongst others).

The prevailing linear economy of synthetic rubber-based materials raises serious sustainability concerns due to the utilization of non-renewable feedstocks^1^ and the improper disposal of vulcanized rubber waste^2^. Linear polydienes, including polybutadiene (PBD) or polyisoprene (PI), represent a major class of synthetic rubber that is distinguished by its mechanical robustness^3^, elasticity^4^, and chemical resistance^5^ (Fig. 1). Derived from the polymerization of petroleum-based, conjugated monomers such as 1,3-butadiene^6^, polydienes bear a C=C double bond in each repeating unit. Exploiting the reactivity of this backbone unsaturation^7^, linear rubber chains are cross-linked during vulcanization via mono-, bi-, and polysulfide bonds on the allylic carbon^8^, forming a three-dimensional network that induces tunable mechanical properties such as tensile strength and elasticity^9^. Initially reported by Goodyear in the 19th century^10^, current vulcanization processes first proceed via the formation of reactive complexes, which catalytically accelerate the generation of (poly)sulfide cross-links^11^ (Fig. 2a). The example of car tires illustrates that further improvement in tensile strength, abrasion resistance and wet grip can be achieved by adding nanoparticles such as carbon black, silica, and clays as reinforcing fillers to yield nanocomposites^12^ (Fig. 2b). A tire is a multilayered composite structure in which vulcanized rubber compounds are combined with reinforcing textiles and steel components to provide strength, stability, and airtightness^13^. This complex architecture exemplifies how vulcanized rubber is typically integrated into advanced composite materials. Another strategy to precisely tune material properties is copolymerization, as exemplified by styrene-butadiene rubber (SBR)^14^ and nitrile-butadiene rubber (NBR)^15^ (Fig. 1b). Variation in comonomer identity and composition can modulate the material’s oil resistance, tensile strength, and chemical durability. Hence, due to their versatile material properties, polydienes and their respective copolymers are relevant for a broad scope of applications^16^. In 2024, the size of the synthetic rubber market was valued at 21.47 billion USD with a predicted annual growth rate of 4%, reaching USD 29.38 billion by 2032^17^. As of 2024, butadiene rubber (BR) holds the largest market share of 26.4% due to its widespread utilization in tire production, industrial rubber manufacturing, and sporting equipment^17,18^. However, since vulcanized rubber is cross-linked (Fig. 2) and often a constituent of complex composites, upcycling or downcycling processes and thus valorizing end-of-life (EOL) vulcanized rubber remains inherently challenging^19^. Opposing the ideal hierarchy of waste management options^20^, end-of-life tires (ELTs), which are primarily constituted of vulcanized PBD rubber, are still poorly managed. For instance, in the US, 274 million tires were discarded in 2021, of which 17% or over 1 billion pounds of tire material^21^ were disposed of in landfills. Globally, 42% of ELTs are utilized for material recovery, 29% are collected without a determined end-use (e.g., in China), 15% are used for energy recovery, 12% are either landfilled or stockpiled, and only 2% are repurposed via civil engineering or backfilling^20^ (Fig. 1c). A serious societal challenge arises from this inadequate EOL management: Landfilling or stockpiling of ELTs not only consumes valuable space but also risks direct leaching^22^ of toxic compounds and potentially microplastics into the environment, or even initiates auto-ignition events^23,24^. Furthermore, the release of hazardous substances such as benzene, dioxins, and furans^22^ during material recovery processes (e.g., pyrolysis) causes additional environmental and health risks.

Given the complex nature of vulcanized rubber waste mismanagement, it is crucial to implement robust policy frameworks, such as Extended Producer Responsibility (EPR^25^), intended to hold manufacturers liable for the full product life cycle, along with international guidelines, such as the OECD (Organisation for Economic Co-operation and Development) policy reports^26^ on circular economy strategies and waste-as-resource systems. However, the EOL management of discarded vulcanized rubber materials remains largely unresolved, particularly in light of the continuously increasing production of synthetic rubber^17^. Addressing this issue requires an interdisciplinary approach that relies on chemical innovation and policy development. While the preferred waste hierarchy (Fig. 1d) typically prioritizes chemical recycling over upcycling^20^, the practical implementation of chemical recycling for waste rubber valorization remains severely limited. The cross-linked nature of rubber materials, which in turn complicates depolymerization, poses potential environmental, and health concerns^22^, and imposes high energy demands for chemical recycling^27^. Accordingly, these limitations urge chemists to develop alternative solutions.

In contrast to studies addressing the entire life cycle of polydienes^28^ and complementary to chemical recycling, the present perspective focuses on challenges and opportunities in post-polymerization modification (PPM) of linear and vulcanized BR, highlighting the progress in this field within the last five years. Our discussion focuses on functional upcycling^29^, which can be achieved by exploiting PPM^30^ to introduce functional handles into cross-linked and linear rubber substrates without depolymerization, potentially yielding value-added materials, with particular emphasis on a metrics-aware, design-focused viewpoint that positions PPM as a precision tool for functional upcycling. A complementary, broader overview of rubber upcycling strategies across chemistry classes and application contexts is provided in our recent review^31^.

In this Perspective, we distinguish between polymer modification and upcycling, whereby upcycling requires evidence of increased material value or sustainability benefits, as quantified by life-cycle assessment (LCA) or techno-economic analyses (TEA). Furthermore, we systematically evaluate the alignment of the discussed PPMs with Green and Circular Chemistry principles, highlighting sustainability challenges and research gaps in the field (Fig. 1e). To mitigate the waste rubber crisis, our perspective extends the broader systems-level view of Wu et al.^28^ and provides a targeted chemical roadmap for future research on sustainable linear and vulcanized rubber modification through PPM-enabled functionalization.

### Precision functionalization strategies for linear PBD towards advanced material design

#### Transition metal catalysis as cornerstone for PBD PPM

Transition-metal (TM) catalysis plays a foundational role in the transformation of metal-catalyzed PPM of PBD. Particularly, hydrogenation reactions^32^ have been explored since the early 20th century to modulate polymer properties. While early reports primarily focused on empirical process development, recent advances shifted towards mechanistically guided strategies. An illustrative example of this evolution is the work by Orwat et al.^33^, who systematically evaluated TM catalysts, revealing the effect of metal type, catalyst loading, and ligand presence on the hydrogenation efficiency and selectivity. Beyond hydrogenation, hydroformylation and respective hydrofunctionalization of polydienes date to the 1960s^34^, and extensive progress in metal-catalyzed post-polymerization strategies for polar group incorporation into polydienes (e.g., via hydroformylation) is summarized in a perspective by Rodriguez et al.^35^ More recently, hydrofunctionalization of internal alkenes in PBD advanced with Carreira-type metal-hydride atom transfer (MHAT) catalysis. Yin et al.^36^ improved MHAT efficiency with over a 250-fold-increase in turnover number [TON] and selectivity by using molecular sieves to extend the catalyst lifetime.

**Fig. 3:**
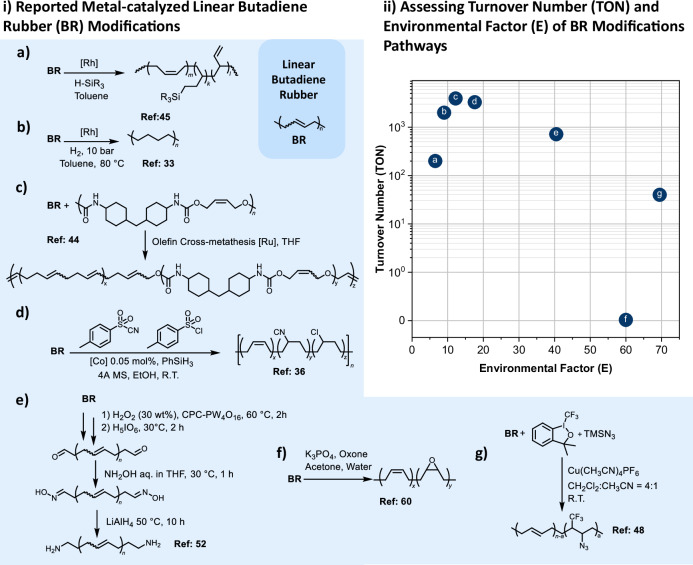
Metal-catalyzed post-polymerization modification strategies for linear butadiene rubber (BR). i Representative reactions: **a** Rh/Pt-catalyzed hydrosilylation of butadiene rubber (BR), Januszewski et al.^45^, **b** Rh/Pt/Ru/Pd-catalyzed hydrogenation, Orwat et al.^33^, **c** Ru-catalyzed olefin cross-metathesis with urethane-functionalized olefins, Shoda et al.^44^, **d** Co-catalyzed metal-hydride atom transfer (MHAT) hydrochlorination, Yin et al.^36^, **e** Epoxidation-reduction sequence yielding amine-terminated *cis*-BR, Xu et al.^52^, **f** continuous-flow dimethyldioxirane (DMDO) epoxidation, Ahlqvist et al.^60^, and **g** Cu-catalyzed azide-trifluoromethylation, Wang et al.^48^. ii Comparative benchmarking of turnover number (TON) versus environmental (E) factor for the above strategies (see section S1 in the Supplementary Information for a detailed explanation of TON and E-factor estimations). The analysis highlights strong contrasts between methods: metathesis and MHAT catalysis achieve high TONs (>3000)^36^ with moderate E factors, hydrogenation^33^ and hydrosilylation^45^ provide balanced but less robust performance, whereas oxidative DMDO^60^ epoxidation shows very low TON (≈0.0133) and high E (≈60), underscoring efficiency gaps in oxidative post-polymerization modification (PPM). Together, these comparisons illustrate how TON/E analysis enables transparent assessment of catalytic robustness and environmental burden across diverse linear BR functionalization pathways.

TM catalysis also enables innovative depolymerization strategies, extending its role from functionalization to controlled macromolecular breakdown. As a representative example, Warner et al.^37^ developed a method using microencapsulated Hoveyda-Grubbs second generation catalyst (HG2) for the in-situ depolymerization of PBD. In a complementary study, Bruening et al.^38^ copolymerized ethylene and butadiene using a titanium catalyst and later applied ruthenium-catalyzed ethenolysis to yield C_10_ to C_20_ α,ω-dienes, industrial precursors typically inaccessible from current olefin processes. Similarly, Burelo et al.^39^ used metathesis with functional chain transfer agents to generate polyesters and polyols from PBD. Building on this, Vos et al.^40^ further optimized hydrogenation-ethenolysis combinations, showing that high-pressure hydrogenation followed by benzoquinone-assisted ethenolysis leads to selective C_4__n+2_ diene formation. To accelerate catalyst development, Cannavacciuolo et al.^41^ employed high-throughput screening and, by evaluating 28 Group 4 metal catalysts for PBD degradation, found Zirconium-based complexes to be the most effective. Taking a distinct approach, Ren et al.^42^ used Ru catalysts supported on monoclinic zirconia to fully cleave C=C and C–C bonds in rubber under mild conditions, producing methane-rich gas. Finally, Foster et al.^43^ repurposed PBD as a macromolecular chain transfer agent in ring-opening metathesis polymerization (ROMP). From a macromolecular design point of view, Shoda et al.^44^ applied cross-metathesis to decorate PBD with olefin-functionalized polyurethanes, yielding urethane-containing polybutadiene (PBUs). A central limitation in comparing different TM catalyzed PPMs of polydienes is the lack of consistent reporting of turnover numbers (TONs; defined as the number of substrate molecules transformed per catalytic site before deactivation) or E factors (defined as the environmental factor quantifying the mass of waste produced per unit mass of product) in the literature, making it difficult to evaluate the catalytic efficiency. Our comparative estimation of TONs and E factors for selected TM-catalyzed PPMs^33,36,44,45^ (Fig. 3 and section S1 in the Supplementary Information) highlights the main differences  between catalytic systems. For instance, the MHAT hydrochlorination of PBD by Yin et al.^36^ achieved a TON of 3300 and an environmental (E)-factor of 18 (Fig. 3ii, d), demonstrating excellent catalyst utilization at very low catalyst loadings (0.05 mol%), with moderate waste generation linked to auxiliary reagents. By contrast, the cross-metathesis approach of Shoda et al.^44^ delivered an even higher TON of 3940 with a lower E-factor of 12 (Fig. 3ii, c). This illustrates how a judicious choice of functional comonomer and reaction design can couple high selectivity with reduced material burden. The hydrogenation system of Orwat et al.^33^ reached a TON of 2000 with an E-factor of 9 (Fig. 3ii, b), reflecting good overall balance, albeit at a slightly higher catalyst loading ([M]:[C=C] = 0.0005mol/mol) compared to the MHAT approach.

#### Click chemistry-based PBD modification

Click chemistry, honored with the 2022 Nobel Prize in Chemistry^46^, exemplifies simplicity, modularity, and orthogonality in chemical synthesis. Tian et al.^47^ demonstrated the power of click chemistry through a two-step PPM strategy: partial hydrogenation of 1,2-enriched PBD followed by thiol-ene modification. In a complementary approach, Wang et al.^48^ introduced an efficient one-step copper-catalyzed azide-trifluoromethylation of PBD. Using Cu(CH_3_CN)_4_PF_6_ and Togni’s reagent (i.e., 1-trifluoromethyl-1,2-benziodoxol-3(1*H*)-one, a novel electrophilic trifluoromethylating reagent), they achieved simultaneous incorporation of –CF_3_ and –N_3_ groups. It is important to note that Wang et al.^48^ report a Togni-reagent-mediated azide-trifluoromethylation that installs azide handles (latent click motifs) and CF₃ groups in a single step with a TON of 40 and E = 70 (Fig. 3ii, g); however, the Togni transformation is stoichiometric, so it does not qualify as a classical click process. The higher E-factor reflects the stoichiometric amount of hypervalent-iodine reagent, ancillary copper salts, and attendant solvent/purification demands. Key research needs to cover: (i) developing solvent-free or intrinsically low-E-factor protocols and catalytic (or metal-free) CF_3_/azide installation methods; (ii) designing removable/recyclable catalysts to raise TON and reduce metal residues; and (iii) exploring one-step, multi-functionalization strategies using orthogonal click sequences with tunable stoichiometry.

#### Silicon-based PBD functionalization

Silicon-based groups play a crucial role in the PPM of PBD by enabling hybrid functionality, interfacial compatibility, and post-functionalization versatility. Januszewski et al.^45^ systematically explored the hydrosilylation of PBD using a diverse set of alkyl-, aryl-, and alkoxysilanes, catalyzed by platinum and rhodium complexes. In follow-up studies, Januszewski and colleagues^49^ demonstrated the design of hybrid materials via Rh(I)-catalyzed additive cross-linking of liquid PBD with a variety of organosilicon reagents. Quantitative benchmarking of the hydrosilylation^45^, however, reveals a TON of only 200 with an estimated E-factor of 7 (Fig. 3ii, a), indicating modest catalyst performance despite favorable material efficiency. Key research gaps include: (i) replacing precious metals (Pt, Rh) with earth-abundant or metal-free alternatives (ii) systematic reporting of TON, turnover frequencies (TOF), and recyclability, currently underexplored in hydrosilylation; and (iii) strategies for multifunctionalization in a single step with tunable Si-loading.

#### Nitrogen-based functionalization

Nitrogen-containing groups offer another versatile functionalization vector for PBD PPM due to their ability to confer ionic conductivity, intermolecular bonding, and network tunability, making them especially valuable in applications ranging from membranes to elastomers. In this context, Laskowski et al.^50^ introduced pendant amine groups into PBD via tandem hydroformylation and reductive amination (aminomethylation), catalyzed by a ruthenium complex. In a complementary advance, early TM-catalyzed hydroaminoalkylation has been demonstrated as a powerful single-step method to introduce amines directly into PBD^51^. Using a tantalum catalyst, aryl and alkyl secondary amines were coupled to both 1,2- and 1,4-polybutadiene segments. In a stereoselective approach, Xu et al.^52^ developed a method to prepare primary amine-terminated *cis*-polybutadiene with a high *cis*-1,4 content (>96%). Starting from PBD, they introduced aldehyde termini via epoxidation and oxidative cleavage, which were converted into oximes and subsequently reduced to primary amines using Red-Al. Focusing on rheology, Malmir et al.^53^ utilized hydroaminoalkylation to introduce hydrogen-bonding amines along the PBD backbone. From a quantitative standpoint, nitrogen-based functionalization strategies remain underexplored with respect to catalytic benchmarking. The stereoselective route of Xu et al.^52^ to amine-terminated *cis*-PBD (ATPB) achieved a TON of 417 with an E-factor of 40 (for an estimation of the ATPB step see Fig. 3ii and for TON and E-factor estimation see section S1 in the Supplementary Information). Compared to Shoda et al.^44^ cross-metathesis (TON = 3940, E = 12; see Fig. 3ii, c), the approach of Xu showed moderate catalyst robustness, but relatively high waste generation due to multi-step activation and stoichiometric reductants. Progress will require direct, single-step amination strategies with lower reagent burdens and earth-abundant or metal-free systems that combine high TONs with intrinsically low E-factor values.

#### Oxidative functionalization

The oxidative modification of polydienes can be traced back to the mid-19th century, when Spiller^54^ first reported the oxidation of India rubber by nitric acid. Shortly thereafter, further studies on the combined action of light and oxygen on rubber^55^ established oxidation as a defining transformation pathway, laying the groundwork for modern strategies in controlled oxidative functionalization of PBDs. Moreover, drawing on principles established in classical oxidative works, enzyme-mediated systems such as lipoxygenase/linoleic acid or horseradish peroxidase/1-hydroxybenzotriazole have been shown to depolymerize *cis*- and *trans*-1,4-polyisoprenes by generating radical species for chain-scission^56^. For a more recent summary focusing on enzymatic polydiene PPM, we refer to the review of Soares et al. ^57^.

Introducing oxygen-containing functionalities^58^ can endow PBD with grafting points, compatibilizing features, or crosslinking capacity^59^. For instance, Ahlqvist et al.^60^ explored a safe and scalable epoxidation method using dimethyldioxirane (DMDO), a short-lived, highly selective oxidant generated in situ from oxone and acetone, which was generated in situ via a continuous-flow stirred tank reactor (CSTR). Accordingly, the method was successfully applied to unsaturated polyolefins, including PBD, to yield epoxidized polymers that are suitable for post-modification. Pushing applications further, Januszewski et al.^61^ demonstrated how oxidized and silylated PBD derivatives can act as precision toughening agents in commercial epoxy thermosets. Despite its long history, oxidative functionalization of polydienes is rarely benchmarked using quantitative metrics. For instance, Ahlqvist et al.^60^ achieved scalable DMDO epoxidation under continuous-flow conditions, but with a very low TON of 0.0133 and a high E-factor of 60 (Fig. 3ii, f), underscoring the inefficiency of current systems. Most reported methods still rely on stoichiometric oxidants, produce substantial waste, and lack data on catalyst recyclability or removal. Progress will require catalytic protocols with benign oxidants (O_2_, H_2_O_2_), systematic TON and E reporting, and strategies that couple multifunctionalization with sustainability.

### Metal-free strategies for linear PBD functionalization

**Fig. 4:**
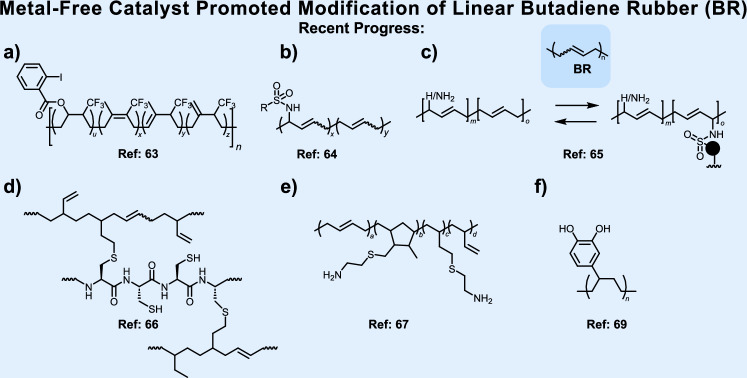
Metal-free post-polymerization modification strategies for linear butadiene rubber. Representative advances include: **a** hypervalent iodine-mediated fluorination, Cao et al.^63^, **b** selenium-catalyzed allylic amination, enabling sulfonamide grafting with tunable properties, Hodges et al.^64^, and **c** reversible sulfamate crosslinking for recyclable elastomers, Hodges et al.^65^. Biologically inspired designs include **d** poly(L-cysteine)-mediated thiol-ene crosslinking for degradable networks, Tsuchiya et al.^66^ and **e** vanillin-derived imine adaptive networks combining reprocessability, self-healing, and shape memory, Yang et al.^67^. Extending towards multifunctional systems, **f** solvent-free Friedel-Crafts alkylation installs catechols for heavy metal remediation, Sehn et al.^69^.

In parallel to metal-catalysis, the motivation to develop safer, greener, and more cost-efficient methods fostered metal-free catalysis as a parallel movement (Fig. 4). The origin of this paradigm shift also lies in the supply chain vulnerabilities impacting e.g., the availability of Pt-based catalysts due to substantial raw material scarcity and geopolitical conflicts^62^.

#### Hypervalent Iodine-mediated functionalization

Among the most promising platforms in this research field are hypervalent iodine(III) reagents, whose oxidative versatility mirrors their growing application in late-stage functionalization of natural products and polymers alike. For example, Cao et al.^63^ demonstrated the selective introduction of fluorinated groups into *cis*-1,4-polyisoprene using hypervalent iodine compounds bearing electron-withdrawing substituents (e.g., CF_3_, C_6_F_5_CO_2_; see Fig. 4a). Follow-up transformations, using acid-induced elimination or azide installation as latent click handle, expanded the chemical landscape and offered modular access to unsaturated and fluorine-rich elastomers. Pushing the boundaries of selective C-H functionalization further, and moving beyond halogen chemistry, Hodges et al.^64^ introduced a selenium-catalyzed approach, for the site-selective allylic amination of PBD without perturbing the alkene configuration (Fig. 4b). Importantly, the resulting materials display finely tunable surface energies and glass transition temperatures, attributes governed by both sulfonamide identity and grafting density. Subsequent work by the same group^65^ unlocked a reversible crosslinking strategy using electrophilic sulfamate handles and diol cross-linkers (Fig. 4c). These networks can be disassembled on-demand via nucleophilic alcoholysis, enabling full material recovery with no loss in macromolecular integrity. This reversible design not only mirrors the adaptive nature of dynamic covalent chemistry but also sets the stage for recyclable rubbers based on selective and catalyst-free network design. Metal-free catalytic strategies based on selenium-mediated allylic amination^64,65^ raise concerns regarding the toxicity and environmental fate of organoselenium compounds, particularly in applications with human or ecological exposure. Despite their innovative nature, these methods frequently lack broad substrate scope or selectivity across different PBD microstructures (*cis*-, *trans*-, or* vinyl*-rich), which limits generalizability. Moreover, catalyst performance is difficult to evaluate, as key parameters such as TON and TOF are rarely reported.

#### *N*-containing crosslinker

Extending the logic of reversibility into biologically derived systems, Tsuchiya et al.^66^ introduced poly(*L*-cysteine) (polyCys) as a thiol-rich polypeptide cross-linker for PBD (Fig. 4d). Upon photo-mediated thiol-ene cross-linking reaction, robust yet hydrolyzable gels were obtained. This design maintains the mechanical integrity of traditional thermosets, while enabling degradation under mildly acidic aqueous conditions. Remarkably, this approach reimagines classical vulcanization, long dominated by sulfur chemistry, as a peptide-guided, degradable alternative aligned with modern biocatalytic ideals and lays the groundwork for biocatalytic rubber technologies. However, degradable crosslinks that enable network deconstruction often compromise tensile strength or thermal resistance, as observed in both the selenium-based^65^ and thiol-ene^66^ approaches. While catalyst-free thiol-ene crosslinking using polyCys^66^ avoids the use of metal catalysts, it may generate substantial aqueous or organic waste, leading to high E-factor values that undermine its Green Chemistry potential. Tackling the recyclability-performance paradox inherent to conventional rubber networks, Yang et al.^67^ reported the synthesis of imine-based covalent adaptive networks (CANs) using a vanillin-derived tripodal cross-linker (Fig. 4e). These imine-based networks, which are formed with 1,2-polybutadiene, enable reprocessing, self-healing, and degradability, without compromising tensile strength. Added functionality, such as shape memory, antibacterial behavior, or weldability, was directly linked to the dynamic Schiff base chemistry.

Pushing PBD repurposing into the realm of energy materials, Fan et al.^68^ developed a solar-thermal sponge by crosslinking PBD with octadecylamine-functionalized reduced graphene oxide (ODA-rGO). The resulting 3D porous structure integrates phase change paraffin materials and exhibits reversible solar-to-thermal energy conversion over >200 cycles, with heat storage capacity exceeding 170 J g^−1^. Importantly, the sponge was fabricated via a self-sacrificial templating process that requires no metal catalysis, yet yields a mechanically robust, thermally dynamic system.

#### Solvent-free alkylation

In a final example of metal-free ingenuity, Sehn et al.^69^ used bulk-phase Friedel-Crafts alkylation to install catechol moieties onto PBD chains (Fig. 4f). The resulting materials exhibited outstanding heavy metal chelation properties, achieving >95% removal efficiencies for Cu^2+^ and Fe^3+^ ions. Conducted under solvent-free conditions at elevated temperature, the one-step protocol provides a rapid and scalable entry point into functional PBD derivatives for water purification and environmental remediation. Catechol-modified elastomers may further serve as redox-active or adhesive bioinspired materials, drawing conceptual inspiration from mussel-inspired adhesive proteins and supramolecular chemistry.

Functionalized PBDs, such as vanillin-modified elastomers^67^, demonstrate strong potential for sustainable material design, yet these systems often suffer from mechanical drawbacks and are not reprocessable, restricting their integration into circular economy models. Furthermore, quantitative sustainability metrics like E-factor, energy input, or toxicity assessment remain scarce, complicating techno-economic evaluations. Only a few studies employ renewable reagents, with vanillin being a rare example^67^, while greener alternatives to conventional oxidants or amines remain largely unexplored. Although advanced functionalities such as self-welding, shape memory, and antibacterial activity have been demonstrated individually, a systematic integration of these features into multifunctional upcycled PBD platforms is still lacking.

### Catalyst-free PPM of PBD

**Fig. 5:**
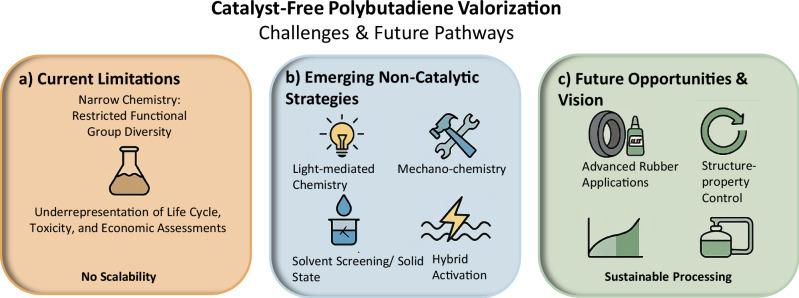
Summary of challenges, innovations, and future directions in non-catalytic strategies for polybutadiene functionalization. **a** Current limitations include restricted functional group diversity, poor selectivity, lack of scalability, and limited life cycle or economic assessments. **b** Emerging non-catalytic strategies such as light-mediated chemistry, mechanochemistry, solvent screening/solid-state approaches, and hybrid activation. **c** Future opportunities lie in enabling sustainable processing, advanced rubber applications, and enhanced structure-property control.

Shifting towards sustainability, catalyst-free routes (Fig. 5) offer a compelling alternative to the intensively tailored catalytic methods long employed in PBD modification. Inspired by the 1995 Nobel Prize in Chemistry, which was awarded for the understanding of uncatalyzed atmospheric reactions concerning ozone depletion^70^, non-catalytic PPM aims for a comparable molecular efficiency, such as transformations driven by inherent reactivity, external stimuli, or benign reagents. In this paradigm, reactions are driven by the substrate itself, light, or inexpensive oxidants, ideally proceeding under mild and solvent-free conditions.

#### Epoxidation as a benchmark catalyst-free PBD modification

Epoxidation, a leading example of this paradigm, is an established strategy for enhancing filler compatibility in rubbers by introducing polar functionalities into nonpolar polymer backbones^58^. While traditional epoxidizing agents such as peracids and hydrogen peroxide are efficient^58^, they often suffer from byproduct formation and operational hazards. In response to these limitations, Cao et al.^71^ explored the use of DMDO for the metal-free epoxidation of low-unsaturation butyl rubber. Through a Design of Experiments (DoE) framework, the group optimized reaction parameters to yield epoxidized rubber with tailored functionality. Crucially, the method retained high efficacy even under aqueous conditions (up to 25 wt% water in the presence of a quaternary ammonium salt), and minimized side reactions typical for ring-opening pathways. Enhanced silica dispersion in modified rubber matrices, proved by rubber process analysis (RPA) and atomic force microscopy (AFM), highlights the relevance of the method for advanced composite design. This study demonstrates how molecular selectivity, process simplicity, and water tolerance can converge in a catalyst-free, green transformation with direct implications for tire and elastomer technology.

#### Catalyst-free functionalization beyond oxidation

Catalyst-free click functionalization has also been realized. Jiang et al.^72^ reported a room-temperature, catalyst-free 1,3-dipolar nitrile N-oxide addition to styrene-butadiene backbones, enabling the introduction of ionic groups under mild conditions. In their study, solution-polymerized poly(styrene-*co*-butadiene) (SSBR) was modified either in solution by refluxing a THF (or THF + 15 wt% DMSO) solution of SSBR with 2-20 mol% nitrile *N*-oxides (CNO-py or CNO-COOH) for 6 h, or in the solid state without any solvent or catalyst by mechanical kneading at 70 °C for 15 min. The reactions achieved 80–97% nitrile *N*-oxide conversion and 2–16% C = C bond functionalization, yielding pyridine- and carboxyl-functionalized SSBRs. The resulting ionically functionalized rubbers exhibited a glass-transition-temperature (*T*_g_) increase from −63 °C (unmodified SSBR) to approximately −10 °C at 16% modification, confirming reduced chain mobility due to grafting. Mechanical testing revealed enhanced tensile strength and elongation at break relative to the parent SSBR, especially when both COOH and pyridine groups were introduced, owing to reversible hydrogen-bond-based cross-linking between the pendant acidic and basic sites. The ability to access ionically functionalized polydienes without catalysts demonstrates that non-oxidative pathways are feasible, significantly expanding the functional space of catalyst-free polydiene modification.

While catalyst-free chemical modification (Fig. 5) offers a promising route to greener rubber functionalization, existing methods remain limited in scope. Developing site-selective reactions that introduce hydroxyl, carboxyl, or amine groups under mild, additive-free conditions continues to be a key challenge. Combining light, heat, and mechanical energy to promote non-catalytic transformations represents another largely untapped frontier. Moreover, the structure-property relationships of modified PBDs are still poorly understood, constraining rational material design for applications such as tires or adhesives.

### Chemical modification of vulcanized rubber

**Fig. 6:**
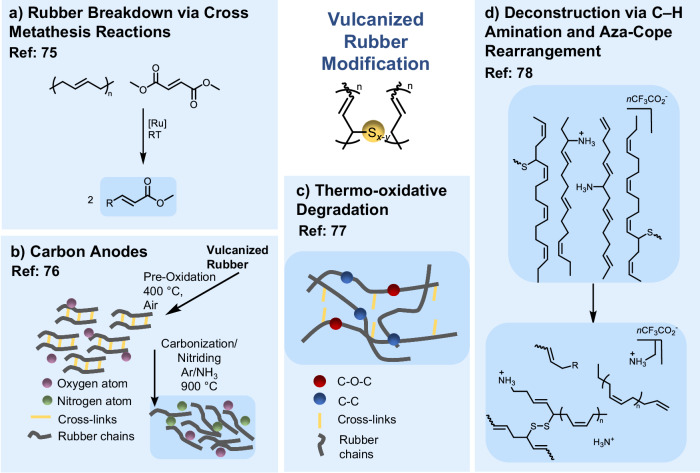
Representative recent strategies for chemical modification of vulcanized polybutadiene-based rubbers. **a** Catalytic disassembly via cross-metathesis: Smith et al.^75^ demonstrated the breakdown of polybutadiene and styrene-butadiene rubber networks through Grubbs-catalyzed cross-metathesis with diesters, yielding soluble low-molecular-weight fragments. **b** Oxidative-nitriding upcycling: Sun et al.^76^ converted waste tires into nitrogen/oxygen-enriched hard carbon materials through sequential pre-oxidation and nitriding, producing high-surface-area carbons for high-performance potassium- and sodium-ion battery anodes. **c** Thermo-oxidative degradation: Yue et al.^77^ examined the temperature-dependent oxidative degradation of vulcanized butadiene rubber, revealing oxygen-diffusion-limited surface oxidation and concurrent chain scission and recombination processes. **d** C–H amination and backbone rearrangement: Towell et al.^78^ developed a sulfur diimide-mediated allylic amination and 2-aza-Cope rearrangement sequence to deconstruct diene polymers and post-consumer rubbers into amine-functionalized oligomers suitable for epoxy resin synthesis.

Chemical modification of vulcanized rubber, as an early example of post-vulcanization chemical treatment, dates back to the late 19th century, when thermal and chemical reclaiming methods were developed to depolymerize or soften scrap vulcanizates for reuse in manufacturing^73^. By the mid-20th century, reclaiming evolved into chemical devulcanization, utilizing alkali digestion, disulfide-cleaving reagents, or metal salts to modify cured rubber networks. This approach laid the foundation for modern post-vulcanization functionalization approaches^74^. Compared with the linear polydiene strategies discussed above, selective functionalization of PBD in vulcanized rubber to yield value-added materials remains challenging due to densely sulfur cross-linked networks that restrict molecular mobility and hinder reagent diffusion, as well as the presence of compounding additives (Fig. 2b). Addressing these limitations, Smith et al.^75^ demonstrated that controlled catalytic pathways can enable reactivity in cured networks (Fig. 6a). Using first or second generation Grubbs’ ruthenium catalysts (G1/G2), they achieved cross-metathesis (CM) of PBD networks with diesters (e.g., dimethyl maleate), catalytically disassembling cross-linked PBD and SBR into soluble fragments. Size-exclusion chromatography (SEC) confirmed a significant molar-mass decrease. The reaction proceeded at ambient temperature in approximately 2.5 h, with diesters accelerating the process as a sufficient catalyst and diester diffused into the SBR matrix to promote network cleavage.

In parallel, Sun et al.^76^ explored oxidative-nitriding transformation routes that convert vulcanized rubber waste into functional carbon materials (Fig. 6b). A two-step pre-oxidation and nitriding protocol yielded nitrogen/oxygen-enriched hard carbon with high surface area and abundant heteroatom doping, delivering excellent anode performance for potassium- and sodium-ion batteries (reversible capacity of 363 mAh g^−1^ after 200 cycles at 100 mA g^−1^) with robust long-term cycling stability. Complementary work by Yue et al.^77^ elucidated the thermo-oxidative degradation of vulcanized BR (Fig. 6c). Between 180–240 °C, oxidation was heterogeneous and oxygen-diffusion-limited, with surface layers forming highly oxidized regions containing new cross-links and oxygen-bearing groups. More recently, Towell et al.^78^ developed a chemical deconstruction approach that enables selective backbone modification and cleavage under mild conditions (Fig. 6d). Using a sulfur diimide reagent, they achieved up to ~35% allylic C–H amination of diene polymers, followed by a cationic 2-aza-Cope rearrangement that deconstructs both model and post-consumer rubbers into low-molar-mass, amine-functionalized fragments. The apparent molecular weight decreased from 5.81 × 10^4^ to ~400 g mol^−1^ after 6 h. The resulting aminated products were subsequently used to fabricate epoxy thermosets with stiffness (i.e., Young’s moduli (*E*) values, with *E* averages of 26.2 MPa) comparable to bisphenol A-derived resins.

### Advancing PBD and vulcanized rubber PPM strategies through green and circular chemistry frameworks

**Fig. 7:**
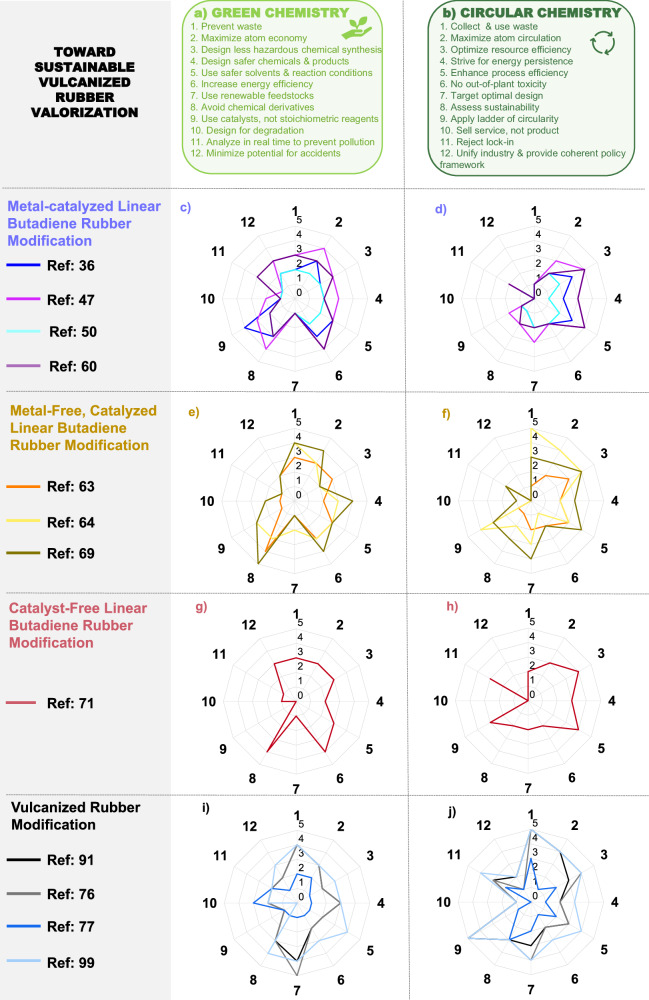
Evaluating linear and vulcanized polybutadiene post-polymerization modification reactions through sustainability frameworks. The scoring system scales from 0 to 5, where 0 indicates non-compliance and 5 indicates full compliance with the investigated principle of the respective framework (see section S2 in the Supplementary Information for a detailed explanation of the estimation of the scores). **a** Green Chemistry principles emphasize waste prevention, atom economy, safer chemicals, and energy efficiency to guide the development of sustainable upcycling processes. **b** Circular Chemistry principles focus on maximizing material circulation, minimizing toxicity, and promoting systemic design for long-term resource retention. **c** Evaluating alignment with Green Chemistry for: Transition-metal catalysis, Yin et al.^36^; Click chemistry, Tian et al.^47^; Aminomethylation, Laskowski et al.^50^; Epoxidation, Ahlqvist et al.^60^. **d** Evaluating alignment with Circular Chemistry for: Transition-metal catalysis, Yin et al.^36^; Click chemistry, Tian et al.^47^; Aminomethylation, Laskowski et al.^50^; Epoxidation, Ahlqvist et al.^60^. **e** Evaluating alignment with Green Chemistry for: Hypervalent iodine-mediated functionalization, Cao et al.^63^; Selenium-mediated amination, Hodges et al.^64^; Aromatic functionalization, Sehn et al.^69^. **f** Evaluating alignment with Circular Chemistry for: Hypervalent iodine-mediated functionalization, Cao et al.^63^; Selenium-mediated amination, Hodges et al.^64^; Aromatic functionalization, Sehn et al.^69^. **g** Evaluating alignment with Green Chemistry for: Catalyst-free epoxidation, Cao et al.^71^. **h** Evaluating alignment with Circular Chemistry for: Catalyst-free epoxidation, Cao et al.^71^. **i** Evaluating alignment with Green Chemistry for: Activated carbon, Murillo et al.^91^; Carbon anodes, Sun et al.^76^; Thermo-oxidative degradation, Yue et al.^77^; Waste rubber composites, Haddaji et al.^99^. **j** Evaluating alignment with Circular Chemistry for: Activated carbon, Murillo et al.^91^; Carbon anodes, Sun et al.^76^; Thermo-oxidative degradation, Yue et al.^77^; Waste rubber composites, Haddaji et al.^99^.

**Fig. 8:**
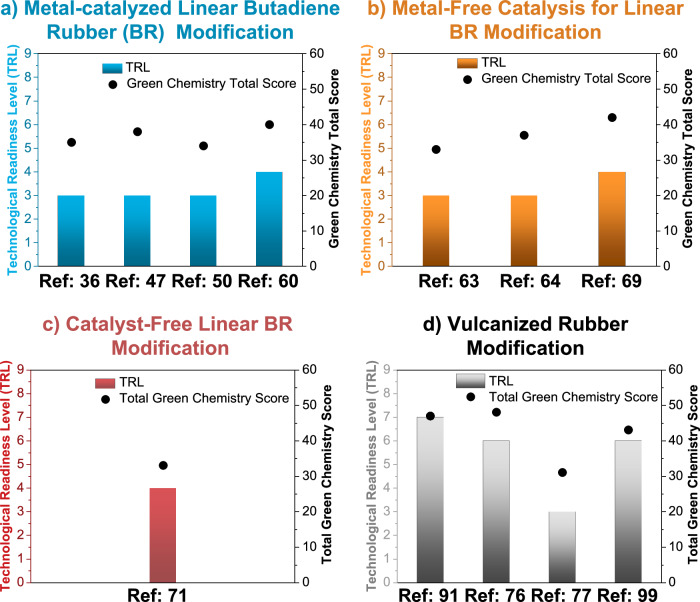
Assessment of technological readiness level. Technological readiness level (TRL, according to the TRL framework of Buchner et al.^83^; see Section S3.3 in the Supplementary Information for a detailed explanation of the estimation of TRL) and total Green Chemistry Score (from 0 to 60, where 60 represents full compliance with all 12 Green Chemistry principles; see Section S3.1 in the Supplementary Information for the calculation of the total Green Chemistry scores) for the following polybutadiene and vulcanized rubber post-polymerization modification reactions: **a** Transition-metal catalysis, Yin et al.^36^; Click chemistry, Tian et al.^47^; Aminomethylation, Laskowski et al.^50^; Epoxidation, Ahlqvist et al.^60^. **b** Hypervalent iodine-mediated functionalization, Cao et al.^63^; Selenium-mediated amination, Hodges et al.^64^; Aromatic functionalization, Sehn et al.^69^. **c** Catalyst-free epoxidation, Cao et al.^71^. **d** Vulcanized rubber modification: Activated carbon, Murillo et al.^91^; Carbon anodes, Sun et al.^76^; Thermo-oxidative degradation, Yue et al.^77^; Waste rubber composites, Haddaji et al.^99^.

Across the diverse strategies explored for linear PBD and vulcanized rubber PPMs, each individual approach demonstrates distinct strengths and limitations in terms of compliance with the principles of Green^79^ and Circular^80^ Chemistry (Fig. 7a, b). To assess this compliance, we assigned scores to exemplary PPM strategies (from 0 to 5, with 0 not being compliant and 5 being fully compliant; see section S2 in the Supplementary Information for a detailed explanation on the estimation of the scores). In particular, TM-catalyzed linear PBD PPM showcases good alignment with the principles of Green Chemistry. This is visually corroborated in the corresponding radar plot in Fig. 7c, which depicts a catalytic efficiency score of 4 / 5 and an atom economy score of 3 / 5 for the metathesis-catalyst-based approach of Yin et al.^36^ Accordingly, metathesis-based PBD PPM offers atom-efficient catalytic transformation pathways, while generating small molecules or telechelic oligomers, which, according to Smith et al.^75^, have the potential to undergo cross-linking reactions due to their PBD-resembling structure (e.g., with benzoyl peroxide (BPO)) and thereby rebuild rubber networks. Additionally, metal-catalyzed linear PBD PPMs exhibit good alignment with the Circular Chemistry framework (Fig. 7d). Still, most TM-catalyzed strategies have been demonstrated only for linear polydienes, which differ markedly from post-consumer vulcanized rubber containing sulfide crosslinks and diverse additives. Thus, despite their conceptual alignment with Green and Circular Chemistry frameworks, the practicality  for these catalytic systems to real waste rubber remains limited. The high catalytic performance of many metathesis catalysts is often hindered by their inherent sensitivity towards sulfur^81,82^, which is present in waste rubber as (poly)sulfide linkages. Consequently, future work should prioritize the development of sulfur-tolerant catalytic systems that can operate directly on post-consumer vulcanized materials. In parallel, the technological readiness of such catalytic PPM strategies must be critically assessed. According to the technological readiness level (TRL) scale proposed by Buchner et al.^83^ (Fig. 8 and Section S3 in the Supplementary Information), the TM-catalyzed hydrofunctionalization reported by Yin et al.^36^ corresponds to TRL 3, where only proof-of-concept experiments validate the feasibility of the reaction. For reference, TRL 1 represents a conceptual idea, while TRL 9 indicates commercial implementation. Although the approach is not yet ready for industrial application, its Green Chemistry score of 35 / 60 (Fig. 8a) highlights its significant sustainability potential.

Similar to transition-metal (TM) catalysis, click chemistry^47^ shows a high score in Green Chemistry metrics because of its selectivity, high atom economy, and minimal need for derivatization (Fig. 7c). For instance, the thiol-ene-based approach reported by Tian et al.^47^ achieves a total Green Chemistry score of 38 / 60 (Fig. 8a). These reactions typically proceed under mild conditions, offering a modular platform to introduce new functionalities. However, they often rely on non-green organic solvents and hazardous reagents like azides or thiols. Still, click chemistry^47^ shows good alignment with Circular Chemistry (Fig. 7d).

Applying the thiol-ene strategy of Tian et al.^47^ to post-consumer vulcanized rubber is hindered by the cross-linked, filler-rich, and chemically aged structure, which limits reagent access and depletes reactive vinyl sites. Impurities such as sulfur, metals, and antioxidants can quench radicals and reduce the reaction efficiency and uniformity. Overall, the approach remains at a low technological readiness (TRL 3), representing only a laboratory-scale proof of concept. Scoring lower than the previous approaches, amine- and nitrogen-containing functionalization^44,50,53^ (e.g., total Green Chemistry score of 34 / 60 for aminomethylation of linear PBD^50^; Fig. 8a) improves PBD reactivity, but the processes often involve potentially corrosive or toxic precursors. Moreover, these reactions rarely imply renewable reagents, and the final products are not typically designed for degradation, reducing their alignment with Green and Circular Chemistry goals (Fig. 7c, d).

The homogeneous Ru hydroformylation catalyst (PICy)Ru(CO)₅^50^ used for aminomethylation of linear PBD is highly sensitive to sulfur and other impurities commonly found in waste rubber, leading to rapid deactivation. Ultimately, the approach remains at a low technological readiness (TRL 3), representing only a laboratory-scale proof of concept (Fig. 8a).

Offering good alignment with the Green Chemistry framework, direct epoxidation strategies, such as those reported by Ahlqvist et al.^60^ yield a total Green Chemistry score of 40 / 60 (Fig. 8a). However, they often involve hazardous oxidants and the metal catalysts may raise concerns regarding toxicity and resource sustainability. This evaluation is further contextualized in Fig. 7c, d. Applying the continuous DMDO epoxidation method^60^ to post-consumer vulcanized rubber faces major barriers due to the presence of residual sulfur compounds, oils, and metal oxides in waste rubber, which could rapidly decompose or quench DMDO, leading to uncontrolled oxidation. Although the process has advanced to TRL 4 (Fig. 8a) through continuous-flow validation and initial process modeling, its application to complex, contaminated rubber feedstocks remains highly limited.

In general, metal-free strategies for linear PBD PPM (Fig. 7e, f) align similarly well with the Green and Circular Chemistry framework as metal-catalyzed PPMs (Fig. 7c, d). Still, metal-free strategies remain limited by the use of non-renewable reagents, hazardous solvents, and relatively low emphasis on catalytic efficiency. Additionally, none of the approaches incorporates service-based business models, such as Michelin’s product-as-a-service concept^84^ (implementing that customers pay per kilometer driven, while the manufacturer retains ownership and responsibility for tire maintenance) or demonstrates clear alignment with policy frameworks (e.g., EPR^25^). Applying the hypervalent iodine-based functionalization of *cis*-1,4-polyisoprene^63^ to post-consumer vulcanized rubber would likely lead to rapid consumption of the hypervalent iodine species due to the presence of sulfur compounds, metal oxides, and antioxidants commonly found in waste rubber. Consequently, this chemistry remains at a low technological readiness (TRL 3, Fig. 8b). As a complementary approach to both metal catalyzed and metal-free linear PBD PPM strategies, the catalyst-free epoxidation of butyl rubber using oxone/acetone, as reported by Cao et al.^71^, demonstrates a slightly higher alignment (total Green Chemistry score of 44 / 60, Fig. 8c) than exemplary metal catalyzed strategies, such as the hydrofunctionalization PPM by Yin et al.^36^ which shows a total Green Chemistry score of 35 / 60 (Fig. 8a). On the one hand, the catalyst-free method reported by Cao et al.^71^ exhibits high atom economy (scoring 3 / 5 for the 2nd principle of Green Chemistry), energy efficiency (scoring 4 / 5 for the 6^th^ principle of Green Chemistry), and minimal derivatization steps (scoring 4 / 5 for the 8^th^ principle of Green Chemistry), without implying metal catalysts or harsh reaction conditions (Fig. 7e). On the other hand, the use of acetone, a flammable solvent, and oxone as a stoichiometric oxidant instead of a catalyst, slightly limits the fulfillment of Green Chemistry principles (Fig. 7e). From a Circular Chemistry standpoint, catalyst-free epoxidation of PBD reflects decent alignment with principles related to waste valorization, atom circulation, and process efficiency (Fig. 7f). Applying the oxone/acetone catalyst-free epoxidation method^71^ to post-consumer vulcanized rubber is significantly constrained by the potential decomposition of oxone and the technological readiness remains low (TRL 3, Fig. 8c). Ultimately, the aim of advancing sustainable linear PBD PPM strategies also holds the promise of aiding the development of versatile and sustainable vulcanized rubber modification pathways. In this context, an exemplary selection of several vulcanized waste rubber modification approaches^76,85–88^ shows the efficient valorization of EoL rubber into functional materials (Fig. 7i, j). Nevertheless, these approaches often rely on energy-intensive processing and lack LCA or integration into broader circular systems (Fig. 7i, j). In particular, the production of hard carbon anodes^76^ from waste tires stands out because it showcases the highest Green Chemistry score of 48 / 60 (Fig. 8d). Although this tire-to-hard-carbon approach has achieved TRL 6 (Fig. 8d) with pilot-scale carbonization, battery testing trials, and prospective demonstration-scale integration, its application to post-consumer vulcanized rubber still demands extensive pretreatment (cleaning, acid washing) to ensure consistent carbon quality. Moreover, variability in tire composition across sources can impact the reproducibility and scalability of battery-grade carbon, highlighting the need for careful feedstock control.

As a complementary example, the production of activated carbon from tires^89^ also demonstrates high alignment with waste utilization requirements (score of 4 / 5 for the 1st principle of Green Chemistry; Fig. 7i) and the direct conversion of waste is in line with reducing derivatives (score of 3 / 5 for the 8th principle of Green Chemistry; Fig. 7i). However, the high temperature activation is energy-intensive, leading to a lower score of 2 / 5 for the 6th principle of Green Chemistry (Fig. 7i). Finally, rubber valorization via a thermo-oxidative degradation approach^90^ ranks lowest among the surveyed methods (with a total Green Chemistry score of 31 / 60; Fig. 8d), reflecting the limited material recovery and higher environmental impact of thermal processing without targeted material design or reuse (Fig. 7i, j). To support the development of sustainable vulcanized rubber valorization strategies, future studies should envision the use of renewable feedstocks, reduce the application of catalysts and solvents and systematically implement LCA, in line with business model innovation^84^ and alignment with emerging policy frameworks^25^. In case catalysts are necessary, their recyclability, efficiency (e.g., TON), cost-efficiency and robustness (e.g., application in the presence of sulfur) must be optimized. Finally, to fully align with the ambitions of Green and Circular Chemistry frameworks, further optimization in terms of process design, energy use, and imparting EoL options by design is crucial. Despite the numerous strategies for valorizing EoL rubber into functional materials, none of them currently achieves true circularity because post-consumer vulcanized rubber cannot yet be fully reprocessed into virgin-quality elastomers. Approaches such as tire-to-hard-carbon anodes^76^ or activated carbon production^91^ promote waste utilization and atom circulation but remain fundamentally linear, converting rubber into alternative materials rather than closing the loop within the rubber lifecycle.

### Future perspective on circular elastomer valorization

Circular elastomer valorization represents the next frontier in sustainable polymer chemistry, aiming to transform EoL rubber into high-value materials through integrated, metrics-driven approaches that unite mechanochemistry, catalysis, and data-guided process design. Mechanochemistry offers an underexplored platform for sustainable PBD PPM, enabling solid-state transformations without the need of solvents, high temperatures, or precious metal catalysts. This approach can improve atom economy, energy efficiency, and scalability while aligning with Green Chemistry principles^92,93^. Integrating mechanochemical processing with LCA and circular design strategies can further strengthen the environmental profile of rubber recycling technologies.

Machine learning (ML) can accelerate sustainable upcycling by predicting reaction outcomes^94^, selectivity, and degradation behavior, thereby optimizing reaction conditions, feedstock preprocessing, and formulation strategies for heterogeneous, additive-rich rubber. Coupled with LCA,^95^ ML-driven workflows can minimize environmental impact and maximize resource efficiency^96–98^.

Despite advances in catalytic, metal-free, and catalyst-free PBD functionalization, industrial translation is constrained by substrate heterogeneity, crosslinking, additives, energy demands, toxicity, and the lack of robust LCA or TEA. Priorities for future research should focus on (i) function-first chemical design, targeting application-relevant properties with renewable and environmentally benign material sources, (ii) scalable, low-impact processing, leveraging mechanochemistry, continuous flow, and light-driven transformations that are compatible with real waste streams, and (iii) data-driven optimization, integrating ML with sustainability metrics to guide rational process design. Realizing a circular elastomer economy will require moving beyond reaction-centric innovation and towards interdisciplinary, metrics-driven platforms that transform PBD from waste into a renewable resource within a closed-loop material landscape.

## Supplementary information

Supplementary Information

## Data Availability

All data are available from the corresponding author upon request
