# Supplementary material for: From waste rubber to value polybutadiene modification for circular materials
Source: Nat Commun. 2026 Feb 5;17:1487. doi: 10.1038/s41467-026-69032-9 (PMC12886778; doi:10.1038/s41467-026-69032-9)
Supplement: Supplementary file 1 — Supplementary Information [file 41467_2026_69032_MOESM1_ESM.pdf]

# Supplementary Information

## From Waste Rubber to Value Polybutadiene Modification for Circular Materials

Janna Jeschke,[1] Hatice Mutlu[1,2,3]

**1.Institut de Science des Matériaux de Mulhouse (IS2M), UMR 7361, Université de Haute Alsace (UHA), Mulhouse, France**

Janna Jeschke, Hatice Mutlu

**2.Department of Chemistry, Rheinland-Pfälzische Technische Universität Kaiserslautern-Landau (RPTU), Technical Polymer Chemistry, Kaiserslautern, Germany**

Prof. Dr. rer.nat. Hatice Mutlu

**3. Leibniz-Institut für Verbundwerkstoffe GmbH, Erwin-Schrödinger-Straße 58 67663 Kaiserslautern, Germany**

Prof. Dr. rer.nat. Hatice Mutlu

Correspondence to: Hatice Mutlu hatice.mutlu@rptu.de

## Content


S1 Calculation of E Factor and TON (Supporting Figure 3) ..... 3

a) Transition metal-catalyzed hydrosilylation of polybutadiene [1] ..... 3

b) Efficient Transformation of Polybutadienes to Polyolefins [2] ..... 3

c) Polybutadiene rubbers with urethane linkages prepared by a dynamic covalent approach for tire applications [3] ..... 3

d) Modifying commodity-relevant unsaturated polymers via Co-catalyzed MHAT [4] ..... 4

e) Modification of polybutadiene with trifluoromethyl and clickable azide groups in one shot [5] ..... 4

f) Continuous dimethyldioxirane generation for polymer epoxidation [6] ..... 4

g) Preparation of primary amine-terminated polybutadiene from cis-polybutadiene [7] ..... 6

S2 Supporting Info Figure 7 ..... 6

S2.1 Metal Catalysis ..... 6

S.2.1.1 Co-catalyzed MHAT modification of unsaturated polymers [4] ..... 6

S.2.1.2 Sequential thiol-ene click and ring-opening polymerization to make functionalized poly(1-butene) [8] ..... 8

S.2.1.3 Aminomethylation of polybutadiene revisited - Nitrogen-based functionalization [9] ..... 9

S.2.1.4 Continuous dimethyldioxirane (DMDO) generation for polymer epoxidation [6] ..... 10

S2.2 Metal-free Catalysis ..... 11

S.2.2.1 Hypervalent iodine functionalization of cis-1,4-polyisoprene [10] ..... 11

S.2.2.2 Selenium-mediated allylic amination for upcycling polybutadiene [11] ..... 12

S.2.2.3 One-step synthesis of catechol-containing polymers via Friedel-Crafts alkylation - use for water decontamination [12] ..... 13

S2.3 Catalyst-Free ..... 15

S2.3.1 Fast, efficient, catalyst-free epoxidation of butyl rubber using Oxone/acetone [13] ..... 15

S.2.4 Vulcanized Rubber ..... 16

S.2.4.1 Activated carbons from waste tire [14] ..... 16

S.2.4.2 Waste tires - hard carbon anodes for K/Na-ion batteries [15] ..... 17

S.2.4.3 Thermo-oxidative degradation behavior of vulcanized butadiene rubber under thermal recycling conditions [16] ..... 18

S.3.4.4 Behavior of waste tire rubber composites reinforced with waste fibers (2025) [17] ..... 20

S3 Supporting Info Figure 8 ..... 21

S3.1 Total Green Chemistry Scores ..... 21

S3.2 TRL Scale for Chemistry [18] ..... 22

S3.3 Recalculated TRL via the Buchner chemistry-TRL scale [18] ..... 23

# S1 Calculation of E Factor and TON (Supporting Figure 3)

## a) Transition metal-catalyzed hydrosilylation of polybutadiene [1]

**Assumptions for E Factor Calculation**

- 100% yield
- H-$SiR_3$ reacts quantitatively
- Catalyst neglected

$$\boldsymbol{E} = \frac{V(toluene)*d(Toluene)}{m(product)} = \frac{9{,}4*0{,}86}{\left((0{,}2309*10^{-3})*5367\right)} = \mathbf{6.52}$$

$$m(product) = n(product) * Mn(PB5)$$

**TON calculated for binuclear Rh(I) catalyst: (10% Si-reagent used relative to C=C)**

$$TON = \frac{moles\ of\ product\ or\ substrate\ consumed}{moles\ of\ catalyst}$$

$$\boldsymbol{TON} = \frac{0{,}1 * 0{,}0185}{0{,}00925 * 10^{-3}} = \mathbf{200}$$

## b) Efficient Transformation of Polybutadienes to Polyolefins [2]

**Assumptions for E factor calculation**

- No $H_2$ and catalyst included
- Product HPB1 considered
- *m*(product) approximated with 1 g

$$E = \frac{m\ (Toluene)}{m\ (product)} = \frac{9}{1} = \mathbf{9}$$

**Calculation of TON for [RhCl($PPh_3$)$_3$] + $PPh_3$**

[Rh] : [C=C] (mol:mol), 0.0005

$$\boldsymbol{TON} = \frac{moles\ (C=C\ consumed)}{moles\ of\ catalyst} = \frac{4431/54}{\frac{54}{\frac{1}{0{,}0005}}} = \mathbf{2000}$$

## c) Polybutadiene rubbers with urethane linkages prepared by a dynamic covalent approach for tire applications [3]

$$\boldsymbol{E} = \frac{\left(V(THF)*d(THF)\right) + m(cat) + m(THP) + \left(V(TEA)*d(TEA)\right) + \left(V(MeOH)*d(MeOH)\right) + \left(V(DCM)*d(DCM)\right)}{m(product)}$$

$$= \frac{(300*0.887) + (0.0372) + (0.53) + (1.52*0.726) + (50*0.791) + (50*1.33)}{30.6} = \mathbf{12.22}$$

Reported yield of 78.8% (used for calculation of TON below):

$$TON = \frac{moles\ (C = C\ consumed)}{moles\ of\ catalyst} = \frac{0.2 * 0.788}{0.04 * 10^{-3}} = \mathbf{3940}$$

d) Modifying commodity-relevant unsaturated polymers via Co-catalyzed MHAT [4]

**Assumptions for E factor calculation:**

- 100% yield
- No catalyst included
- Work-up not included in calculation

$$E = \frac{m(TsCl) + m(EtOH) + m(Phenylsilane)}{n(product) * M(product)} = \mathbf{17.60}$$

**TON was reported in their article**

e) Modification of polybutadiene with trifluoromethyl and clickable azide groups in one shot [5]

$$\boldsymbol{E} = \frac{\left(V(DCM) * d(DCM)\right) + \left(V(ACN) * d(ACN)\right) + m(fluo.agent) + m(cat.) + m(TMSN3)}{m(product)}$$

$$= \frac{(16 * 1.33) + (4 * 0.785) + (261 * 1.62 * 10^{-3}) + (0.054 * 10^{-3} * 372.72) + m(2.16 * 10^{-3} * 73)}{0.3584}$$

$$= \mathbf{69.51}$$

$$TON = \frac{moles\ of\ product\ or\ substrate\ consumed}{moles\ of\ catalyst} = \frac{0.3584/165}{0.054 * 10^{-3}} = \mathbf{40}$$

f) Continuous dimethyldioxirane generation for polymer epoxidation [6]

**Assumptions:**

Experimental details for the 20-min collection (based on SI):

- Polymer solution: **10 g L$^{-1}$** low MW PBD in ethyl acetate.
- Polymer-solution flow (from the 3:1 DMDO:polymer statement): total flow = 1.00 mL min$^{-1}$; polymer solution flow = 1/4 of total = 0.25 mL min$^{-1}$. → in 20 min polymer solution volume = 0.25 mL/min × 20 min = 5.0 mL. Polymer mass supplied = 10 g L$^{-1}$ × 5.0 mL = 0.05 g (this matches stated theoretical 0.05 g from SI, section 5.1). Actual isolated product = 0.0445 g (cf. SI, section 5.1).
- DMDO stream flow = 3 × polymer flow = 0.75 mL min$^{-1}$. Assuming that DMDO was generated by mixing two equal upstream streams (an aqueous oxone stream and a base/acetone stream). This is consistent with the procedure text in the SI where oxone and a base/acetone stream are combined to form DMDO. So the DMDO flow is split into two equal reagent streams of 0.375 mL min$^{-1}$ each:
    - Oxone aqueous stream: 0.375 mL/min → 7.5 mL over 20 min (all water). Oxone concentration used earlier in SI = 0.8 M, so estimation that Oxone mass consumed.

    - $K_3PO_4$ solution (70:30 water:acetone, 0.33 M $K_3PO_4$, as in SI): 0.375 mL/min → 7.5 mL over 20 min. This solution is 70% water / 30% acetone by volume, so acetone in this stream = 0.30×7.5 = 2.25 mL.
- Densities used: ethyl acetate 0.897 g $mL^{-1}$, acetone 0.784 g $mL^{-1}$, water 1.00 g $mL^{-1}$.
- Polymer repeating unit: polybutadiene repeat = $C_4H_6$, Mw ≈ 54.09 g $mol^{-1}$ (one C=C per repeat). They reported 4% epoxidation of polymer = interpreted that as 4% of repeat units were converted to epoxide.

Masses used in 20 min run

- Ethyl acetate (polymer solution): 5.0 mL × 0.897 g $mL^{-1}$ = 4.485 g
- $K_3PO_4$ solution (70:30): total 7.5 mL → acetone 2.25 mL (mass = 2.25 × 0.784 = 1.764 g) and water 5.25 mL (mass = 5.25 g)
- Oxone aqueous: 7.5 mL water = 7.5 g
- $K_3PO_4$ mass: concentration 0.33 M × 0.0075 L = 0.002475 mol; Mw $K_3PO_4$ ≈ 212.27 g $mol^{-1}$ → mass = 0.525 g
- Oxone mass: 0.8 M × 0.0075 L = 0.006 mol; using Mw ≈152.14 g $mol^{-1}$ → mass ≈ 0.913 g
- Product isolated = 0.0445 g

Total mass of inputs (solvents + reagents + product) ≈ 4.485 (EA) + 1.764 (acetone) + 12.75 (total water = 7.5 + 5.25) + 0.525 ($K_3PO_4$) + 0.913 (Oxone) + 0.0445 (product) = ≈ 20.482 g

Mass of waste = total inputs − product = 20.482 − 0.0445 = ≈ 20.437 g

**E-factor (with solvent)** = mass of waste / mass product = 20.437 / 0.0445 ≈ **459**.

**TON calculation for $K_3PO_4$**

- Moles of repeat units in isolated product: 0.0445 g ÷ 54.09 g $mol^{-1}$ = **$8.227\times10^{-4}$ mol**.
- Moles of epoxide formed (4% of repeat units): $8.227\times10^{-4}$ × 0.04 = **$3.291\times10^{-5}$ mol** epoxide.
- Moles $K_3PO_4$ supplied (20 min run at 0.33 M, 7.5 mL) = **0.002475 mol**.
- **TON =** moles epoxide / moles $K_3PO_4$ = $3.291\times10^{-5}$ / 0.002475 ≈ **0.0133.**

**E-factor under different conventions**

1. **Practical plant/process view, assuming that aqueous is excluded and 80% recovery of organic solvents**

- Ethyl acetate waste = 20% of 4.485 = 0.897 g
- Acetone waste = 20% of 1.764 = 0.353 g
-
    - $K_3PO_4$ + Oxone = 0.525 + 0.913 = 1.438 g Waste ≈ **2.688 g → E = 2.688 / 0.0445 ≈ 60.4**

- **If aqueous solvents are excluded** and **substantial solvent recovery assumed** for organics, for example, with 80% recovery of organic solvents (a realistic ballpark for some processes) the E-factor becomes **≈60**

### g) Preparation of primary amine-terminated polybutadiene from cis-polybutadiene [7]

**Assumptions for E factor calculation**

- Starting weight (4 g) approximatively equal to *m*(product)

$$\boldsymbol{E} = \frac{\left(V(Dichloroethane) * d(Dichloroethane)\right) + \left(V(nhexane) * d(nhexane)\right) + m(cat.) + (V(THF) * d(THF))}{m(product)}$$

$$\frac{(80 * 1.25) + (40 * 0.655) + 0.2 + (40 * 0.887)}{4} = \mathbf{40.47}$$

**Exemplary TON for the step of preparing ATPB from EPBD solution was calculated**

$$TON = \frac{moles\ of\ product\ or\ substrate\ consumed}{moles\ of\ catalyst}$$

$$\boldsymbol{TON} = \frac{4.0/54}{0.2/1935} = \mathbf{716}$$

# S2 Supporting Info Figure 7

**Scoring legend to quantify the alignment with each principle of Green and Circular Chemistry:**

- Green / Circular: 0 = none / strongly negative, 1 = very poor, 2 = poor, 3 = moderate, 4 = good, 5 = excellent / exemplary.

## S2.1 Metal Catalysis

### S.2.1.1 Co-catalyzed MHAT modification of unsaturated polymers [4]

**A. Green Chemistry (Anastas & Warner)**

1. **Prevention:** 2 - Method focuses on new product creation, no explicit waste prevention.
2. **Atom Economy:** 3 - Catalytic transformation helps atom economy vs. multistep derivatization but additives/side-products exist.
3. **Less Hazardous Chemical Syntheses:** 2 - Uses cobalt (toxic) and other potentially hazardous reagents.

4. **Designing Safer Chemicals:** 2 - Objective is functionality, no inherent safety or reduced hazard of products.
5. **Safer Solvents & Auxiliaries:** 3 - Typical lab solvents used; no specific safety hazards due to solvents and auxiliaries; however, not particularly hazard avoiding.
6. **Energy Efficiency:** 3 - Reactions reported under moderate conditions; not explicitly energy-optimized.
7. **Use of Renewable Feedstocks:** 1 - Commodity unsaturated polymers are petrochemical-derived; renewables not emphasized.
8. **Reduce Derivatives:** 3 - Direct functionalization reduces synthesis steps compared to protection/derivatization routes.
9. **Catalysis:** 4 - Catalytic MHAT is central (positive), though choice of metal reduces green score.
10. **Design for Degradation:** 1 - No design-for-degradability reported.
11. **Real-time Analysis for Pollution Prevention:** 1 - Not reported.
12. **Inherently Safer Chemistry for Accident Prevention:** 2 - Metal handling and reagents pose risks; no special mitigation discussed.

**B. Circular Chemistry**

1. **Collect & Use Waste:** 1 - Study uses virgin commodity polymers, not waste feedstock.
2. **Maximize Atom Circulation:** 2 - Catalysis improves conversion but no closed-loop atom circulation strategy.
3. **Optimize Resource Efficiency:** 3 - Catalytic approach improves resource efficiency vs stoichiometric routes.
4. **Strive for Energy Persistence:** 2 - Not optimized for energy re-use/low-energy processing.
5. **Enhance Process Efficiency:** 3 - Selective catalysis improves process efficiency.
6. **No Out-of-Plant Toxicity:** 2 - Use of cobalt and potential downstream hazards lowers the score.
7. **Target Optimal Design:** 2 - Focus on product functionality rather than circular-design-for-reuse.
8. **Assess Sustainability:** 1 - Minimal lifecycle or sustainability assessment included.
9. **Apply Ladder of Circularity:** 1 - Research is modification-centric, not focused on reuse/recycling ladder.
10. **Sell Service, Not Product:** 0 - Not applicable.
11. **Reject Lock-in:** 1 - Metal-specific chemistry can create a dependency on particular catalysts.
12. **Unify Industry & Provide Policy Framework:** 0 - Purely academic; no policy/industry unification.

### S.2.1.2 Sequential thiol-ene click and ring-opening polymerization to make functionalized poly(1-butene) [8]

**A. Green Chemistry**

1. **Prevention:** 3 - Click reactions tend to minimize side-products; still generates modified polymer streams.
2. **Atom Economy:** 4 - Click chemistry is more atom economic when compared to many alternatives.
3. **Less Hazardous Syntheses:** 3 - Thiol reagents can be odorous/toxic; conditions are mild but reagent hazard reduces score.
4. **Designing Safer Chemicals:** 3 - The method enables precise functionalization — but safety of new polymers is not necessarily improved.
5. **Safer Solvents & Auxiliaries:** 3 - Common solvents used; not strongly highlighted as safer solvents.
6. **Energy Efficiency:** 4 - Click reactions and ROP often proceed under mild conditions, saving energy.
7. **Use of Renewable Feedstocks:** 1 - Feedstock is petrochemical-derived; no renewables used.
8. **Reduce Derivatives:** 4 - Click methodology greatly reduces protecting-group/deactivation steps.
9. **Catalysis:** 3 - ROP may be carried out under catalytic conditions, whereas click reactions are initiation-driven rather than dependent on continuous catalysis.
10. **Design for Degradation:** 2 - Not a focus (products are functionalized but not necessarily degradable).
11. **Real-time Analysis:** 1 - Real-time analysis was not reported as a central element of the publication.
12. **Inherently Safer Chemistry for Accidents:** 3 - Mild conditions reduce accident risk, but thiol handling is a hazard.

**B. Circular Chemistry**

1. **Collect & Use Waste:** 1 - Work uses virgin monomers/polymers, not wastes.
2. **Maximize Atom Circulation:** 3 - High atom economy supports better atom circulation, but loop not closed.
3. **Optimize Resource Efficiency:** 4 - Efficient reactions and fewer steps improve resource usage.
4. **Strive for Energy Persistence:** 3 - Mild conditions support lower energy input; energy persistence not explicitly designed.
5. **Enhance Process Efficiency:** 4 - Click chemistry is process-efficient and scalable.
6. **No Out-of-Plant Toxicity:** 2 - Some reagents (thiols) and processing solvents raise concerns for downstream toxicity.

7. **Target Optimal Design:** 3 - Allows design of modular functional polymers (useful for product longevity/repair).
8. **Assess Sustainability:** 2 - Limited lifecycle or circularity assessment present.
9. **Apply Ladder of Circularity:** 2 - Enables product design that could favor repair/functionalization, but not explicitly applied.
10. **Sell Service, Not Product:** 0 - Not considered.
11. **Reject Lock-in:** 2 - Method is fairly general but may require specific monomers/chemistry.
12. **Unify Industry & Policy:** 0 - Not addressed.

### S.2.1.3 Aminomethylation of polybutadiene revisited - *Nitrogen-based functionalization* [9]

**A. Green Chemistry**

1. **Prevention:** 2 - Adds new functional groups; not primarily prevention-focused.
2. **Atom Economy:** 2 - Aminomethylation often involves multi-component reagents and byproducts.
3. **Less Hazardous Syntheses:** 2 - Many aminomethylation routes involve formaldehyde/halides/strong reagents.
4. **Designing Safer Chemicals:** 2 - Functionality may improve properties but not inherently safety-focused.
5. **Safer Solvents & Auxiliaries:** 2 - Typical solvents and auxiliaries likely used; no special measures described.
6. **Energy Efficiency:** 2 - Standard lab conditions; not heavily optimized for low energy.
7. **Use of Renewable Feedstocks:** 1 - Feedstock likely petrochemically derived.
8. **Reduce Derivatives:** 3 - Post-polymerization modification reduces need for complex monomer synthesis.
9. **Catalysis:** 2 - Many aminomethylation methods are stoichiometric; catalysis may or may not be used.
10. **Design for Degradation:** 1 - No focus on degradability.
11. **Real-time Analysis:** 1 - Not typical for conference reports unless specifically indicated.
12. **Inherently Safer Chemistry for Accidents:** 2 - Use of amination reagents presents hazards.

**B. Circular Chemistry**

1. **Collect & Use Waste:** 1 - Uses virgin polymer as substrate; not waste-sourced.
2. **Maximize Atom Circulation:** 2 - Adds heteroatoms but does not close loops.
3. **Optimize Resource Efficiency:** 2 - Improves polymer functionality but not resource-circulation focused.

4. **Strive for Energy Persistence:** 1 - Not designed for energy persistence.
5. **Enhance Process Efficiency:** 2 - Post-polymerization modification can be efficient but method-dependent.
6. **No Out-of-Plant Toxicity:** 2 - Potential for toxic reagents/byproducts.
7. **Target Optimal Design:** 2 - Enhances polymer properties but not explicitly for circular end-of-life.
8. **Assess Sustainability:** 1 - Conference talk unlikely to contain full LCA.
9. **Apply Ladder of Circularity:** 1 - No explicit circularity ladder application.
10. **Sell Service, Not Product:** 0 - Not applicable.
11. **Reject Lock-in:** 1 - Could create dependency on specific chemistries for functionalization.
12. **Unify Industry & Policy:** 0 - Not addressed.

### S.2.1.4 *Continuous dimethyldioxirane (DMDO) generation for polymer epoxidation* [6]

**A. Green Chemistry**

1. **Prevention:** 3 - Continuous-flow DMDO reduces batch residues, lowering waste.
2. **Atom Economy:** 3 - Epoxidation consumes oxygen donor; DMDO can be atom-efficient but generation requires reagents.
3. **Less Hazardous Syntheses:** 3 - Continuous DMDO reduces handling hazards compared to batch; DMDO itself is a strong oxidant.
4. **Designing Safer Chemicals:** 2 - Resulting epoxides may increase reactivity/toxicity; not a specific safety aim.
5. **Safer Solvents & Auxiliaries:** 3 - Flow enables smaller solvent inventories; solvent choice matters.
6. **Energy Efficiency:** 4 - Flow chemistry often increases energy efficiency and heat management.
7. **Use of Renewable Feedstocks:** 1 - Polymer substrates are petrochemical in origin.
8. **Reduce Derivatives:** 3 - Direct epoxidation avoids multi-step derivatization.
9. **Catalysis:** 2 - DMDO is a stoichiometric oxidant rather than a catalyst.
10. **Design for Degradation:** 1 - Not addressed.
11. **Real-time Analysis:** 3 - Flow platforms often more amenable to inline monitoring, though implementation varies.
12. **Inherently Safer Chemistry for Accidents:** 3 - Continuous generation reduces DMDO inventory and improves safety vs batch.

**B. Circular Chemistry**

1. **Collect & Use Waste:** 1 - Focus on functionalization of existing polymers, not waste feedstocks.

2. **Maximize Atom Circulation:** 2 - Epoxidation adds O but does not close atom cycles.
3. **Optimize Resource Efficiency:** 4 - Continuous production and safer handling improve resource efficiency.
4. **Strive for Energy Persistence:** 3 - Flow enhances energy efficiency, some persistence gains.
5. **Enhance Process Efficiency:** 4 - Continuous DMDO is process-efficient and scalable.
6. **No Out-of-Plant Toxicity:** 2 - Oxidant residues and byproducts could be hazardous if not managed.
7. **Target Optimal Design:** 2 - Functionalization aids downstream utility but not explicitly circular design.
8. **Assess Sustainability:** 2 - Process benefits suggest sustainability gains but full LCA not shown.
9. **Apply Ladder of Circularity:** 1 - Not focused on reuse/recycling ladder.
10. **Sell Service, Not Product:** 0 - Not applicable.
11. **Reject Lock-in:** 2 - Adoption of flow DMDO may be broadly applicable (some flexibility).
12. **Unify Industry & Policy:** 0 - No policy/unification effort.

## S2.2 Metal-free Catalysis

### S.2.2.1 *Hypervalent iodine functionalization of cis-1,4-polyisoprene* [10]

**A. Green Chemistry**

1. **Prevention:** 3 - Direct functionalization can reduce multi-step synthesis waste.
2. **Atom Economy:** 3 - Hypervalent iodine reagents are often stoichiometric, lowering atom economy vs catalytic routes.
3. **Less Hazardous Syntheses:** 3 - Avoids metals (good), but hypervalent iodine reagents are oxidizing and may generate iodinated byproducts.
4. **Designing Safer Chemicals:** 2 - Products not necessarily designed for safety.
5. **Safer Solvents & Auxiliaries:** 3 - Standard solvents; metal-free approach is positive.
6. **Energy Efficiency:** 3 - Reactions typically mild; not explicitly optimized for energy.
7. **Use of Renewable Feedstocks:** 1 - Polymer is petro-derived.
8. **Reduce Derivatives:** 4 - One-step direct functionalization reduces extra derivatization steps.
9. **Catalysis:** 1 - Stoichiometric reagents dominate; not catalytic.
10. **Design for Degradation:** 1 - Not addressed.
11. **Real-time Analysis:** 1 - Not central to the report.
12. **Inherently Safer Chemistry for Accident Prevention:** 2 - Oxidizing reagents have inherent risks despite metal-free approach.

**B. Circular Chemistry**

1. **Collect & Use Waste:** 1 - Not focused on using waste feedstocks.
2. **Maximize Atom Circulation:** 2 - Stoichiometric iodination reduces ideal atom circulation.
3. **Optimize Resource Efficiency:** 3 - One-step processes improve efficiency, but reagent stoichiometry affects score.
4. **Strive for Energy Persistence:** 2 - Not energy-persistence oriented.
5. **Enhance Process Efficiency:** 3 - Direct functionalization is process-efficient in terms of synthesis steps.
6. **No Out-of-Plant Toxicity:** 2 - Potential for iodinated effluents and oxidant-derived wastes.
7. **Target Optimal Design:** 2 - Focus on property tuning rather than circular end-of-life.
8. **Assess Sustainability:** 1 - LCA not typical in synthetic methodology papers.
9. **Apply Ladder of Circularity:** 1 - Not explicitly applied.
10. **Sell Service, Not Product:** 0 - Not relevant.
11. **Reject Lock-in:** 2 - Metal-free route reduces lock-in to particular metals, but reliance on stoichiometric reagents creates other dependencies.
12. **Unify Industry & Policy:** 0 - Not addressed.

### S.2.2.2 Selenium-mediated allylic amination for upcycling polybutadiene [11]

**A. Green Chemistry**

1. **Prevention:** 4 - Explicit upcycling of existing polymers prevents waste and adds value.
2. **Atom Economy:** 3 - Allylic amination can be fairly atom-efficient depending on the reagents used.
3. **Less Hazardous Syntheses:** 2 - Selenium compounds raise toxicity concerns for reagents/byproducts.
4. **Designing Safer Chemicals:** 3 - Upcycled products may have enhanced properties; safety profile depends on substitution.
5. **Safer Solvents & Auxiliaries:** 3 – Commonly used solvents; process may be compatible with benign solvents but not always reported.
6. **Energy Efficiency:** 3 - Reactions performed under moderate lab conditions; not explicitly optimized for minimal energy.
7. **Use of Renewable Feedstocks:** 2 - Uses waste polymer feedstock (positive) but monomer origin still petrochemical.
8. **Reduce Derivatives:** 3 - Direct functionalization reduces extra steps.
9. **Catalysis:** 3 - Selenium-mediated approaches can be catalytic or stoichiometric; method-dependent.

10. **Design for Degradation:** 2 - Upcycling typically aims at durable products; degradability not emphasized.
11. **Real-time Analysis:** 1 - Not typically reported.
12. **Inherently Safer Chemistry for Accident Prevention:** 2 - Selenium hazard lowers score despite upcycling benefit.

**B. Circular Chemistry**

1. **Collect & Use Waste:** 5 - Explicit example of *using waste polybutadiene* for upcycling — strong alignment.
2. **Maximize Atom Circulation:** 4 - Upcycling increases useful atom retention relative to degradation.
3. **Optimize Resource Efficiency:** 4 - Adds value to waste streams, improving resource efficiency.
4. **Strive for Energy Persistence:** 2 - Not specifically energy-persistent; may be moderate energy demand.
5. **Enhance Process Efficiency:** 3 - Direct chemical upcycling is reaction step efficient, but reagent hazards complicate upscaling.
6. **No Out-of-Plant Toxicity:** 1 - Selenium species are problematic when released into the environment.
7. **Target Optimal Design:** 3 - Upcycling is a step towards product redesign for longer life/use.
8. **Assess Sustainability:** 2 - Some sustainability rationale present but no discussion of a LCA.
9. **Apply Ladder of Circularity:** 4 - Moves material higher on circular ladder (upcycling vs downcycling).
10. **Sell Service, Not Product:** 0 - Not applicable.
11. **Reject Lock-in:** 2 - Method is specific (selenium chemistry) which can be lock-in for processes.
12. **Unify Industry & Policy:** 0 - No industry/policy integration presented.

### S.2.2.3 One-step synthesis of catechol-containing polymers via Friedel-Crafts alkylation - use for water decontamination [12]

**A. Green Chemistry**

1. **Prevention:** 4 - One-step synthesis reduces waste and streamlines production.
2. **Atom Economy:** 4 - Friedel–Crafts Alkylation can often be atom-economical if well-designed.
3. **Less Hazardous Syntheses:** 2 - Friedel–Crafts Alkylation typically requires Lewis acids (hazardous) and creates acidic wastes.
4. **Designing Safer Chemicals:** 4 - Catechol functionality enables application as remediation material, improving net environmental outcome.

5. **Safer Solvents & Auxiliaries:** 3 - Solvent and reagent choices are considered; while the choices are not exceptional in terms of safety, they are manageable and acceptable.
6. **Energy Efficiency:** 4 - One-step reactions are often energetically favorable when compared to multistep.
7. **Use of Renewable Feedstocks:** 1 - Polymer feedstocks are generally petrochemical based unless otherwise stated.
8. **Reduce Derivatives:** 5 - Efficient one-step synthesis strongly reduces extra derivatization.
9. **Catalysis:** 3 - Friedel–Crafts Alkylation uses catalysts (Lewis acids) but these can be stoichiometric/consumptive.
10. **Design for Degradation:** 2 - Designed for remediation rather than degradability.
11. **Real-time Analysis:** 1 - Not central to the publication.
12. **Inherently Safer Chemistry for Accidents:** 2 - Lewis acids/corrosive reagents reduce safety score.

**B. Circular Chemistry**

1. **Collect & Use Waste:** 3 - Application towards water decontamination can recover resources/clean feedstreams; polymer feedstock origin unclear.
2. **Maximize Atom Circulation:** 3 - Efficient synthesis and pollutant capture support atom/resource recirculation indirectly.
3. **Optimize Resource Efficiency:** 4 - One-step, high-yield synthesis improves resource efficiency.
4. **Strive for Energy Persistence:** 3 - Energy demands likely modest; not explicitly persistence-focused.
5. **Enhance Process Efficiency:** 4 – High process efficiency via one-step synthesis.
6. **No Out-of-Plant Toxicity:** 2 - Lewis acid use and potential leaching from polymer can be problematic.
7. **Target Optimal Design:** 4 - Designing polymers for remediation is a targeted circular-utility design.
8. **Assess Sustainability:** 3 - Application-driven sustainability argument present, but full LCA is typically missing.
9. **Apply Ladder of Circularity:** 3 - Polymers used to remediate/regenerate resources sits mid-ladder (value-adding use).
10. **Sell Service, Not Product:** 1 - Potential for remediation-as-a-service but not part of the paper.
11. **Reject Lock-in:** 2 - Friedel–Crafts conditions can be broadly applied, but reliance on specific catalysts limits flexibility.
12. **Unify Industry & Policy:** 0 - Not addressed.

## S2.3 Catalyst-Free

### S2.3.1 Fast, efficient, catalyst-free epoxidation of butyl rubber using Oxone/acetone [13]

**A. Green Chemistry**

1. **Prevention:** 3 - Catalyst-free route potentially reduces contamination and catalyst-waste.
2. **Atom Economy:** 3 - Oxone (peroxomonosulfate) is a stoichiometric oxidant; atom economy is moderate.
3. **Less Hazardous Syntheses:** 3 - Avoids metals (positive) but generates sulfate byproducts.
4. **Designing Safer Chemicals:** 2 - Epoxidation changes material properties, not necessarily safety-focused.
5. **Safer Solvents & Auxiliaries:** 3 - Acetone is commonly available and relatively benign; Oxone handling requires care.
6. **Energy Efficiency:** 4 - Fast and efficient suggests lower energy input vs. longer processes.
7. **Use of Renewable Feedstocks:** 1 - Butyl rubber is typically petrochemically derived/vulcanized.
8. **Reduce Derivatives:** 4 - Direct, catalyst-free transformation reduces intermediate steps.
9. **Catalysis:** 0 - Catalyst-free by design; positive for avoiding metal catalysts but catalytic benefit absent.
10. **Design for Degradation:** 1 - Not focused on degradability.
11. **Real-time Analysis:** 1 - Not a central topic of the work.
12. **Inherently Safer Chemistry for Accidents:** 3 - Avoiding metal catalysts reduces some hazards but risks from the oxidant remain.

**B. Circular Chemistry**

1. **Collect & Use Waste:** 2 - Can be applied to waste rubber streams, but paper may focus on virgin rubber.
2. **Maximize Atom Circulation:** 3 - Direct epoxidation retains the polymer backbone and adds value, good atom preservation.
3. **Optimize Resource Efficiency:** 4 - Fast, catalyst-free process increases resource efficiency.
4. **Strive for Energy Persistence:** 3 - Fast reactions lower energy use, but no active energy-recovery measures.
5. **Enhance Process Efficiency:** 4 - High process throughput and simplicity are beneficial for circular workflows.
6. **No Out-of-Plant Toxicity:** 2 - Oxone-derived sulfate wastes need treatment to avoid environmental impact.
7. **Target Optimal Design:** 2 - Functionalization for better filler dispersion targets performance not necessarily circular end-of-life.

8. **Assess Sustainability:** 2 - Process-level benefits are evident but LCA is usually absent.

9. **Apply Ladder of Circularity:** 3 - Can enable reuse/upcycling of rubber into higher-value materials.

10. **Sell Service, Not Product:** 0 - Not discussed.

11. **Reject Lock-in:** 3 - Catalyst-free process reduces reliance on catalyst supply chains (positive).

12. **Unify Industry & Policy:** 0 - No policy discussion.

### S.2.4 Vulcanized Rubber

#### S.2.4.1 Activated carbons from waste tire [14]

**A. Green Chemistry**

1. **Prevention:** 4 - Converts waste tire feedstock into useful sorbents; prevents waste from landfills.

2. **Atom Economy:** 3 - Pyrolysis/activation yields char plus gaseous byproducts; atom economy is moderate.

3. **Less Hazardous Syntheses:** 2 - Thermal/chemical activation can release hazardous volatiles; process control is needed.

4. **Designing Safer Chemicals:** 3 - Final activated carbons are benign sorbents with valuable end uses.

5. **Safer Solvents & Auxiliaries:** 2 - Pyrolytic/chemical activation often uses chemicals or high heat, instead of prioritizing safer solvent-based approaches.

6. **Energy Efficiency:** 2 - High-temperature activation is energy-intensive.

7. **Use of Renewable Feedstocks:** 4 - Uses waste (circular feedstock), high marks for feedstock valorization.

8. **Reduce Derivatives:** 3 - Direct conversion of waste to product reduces extra steps.

9. **Catalysis:** 1 - Process is thermally activated rather than catalytic.

10. **Design for Degradation:** 2 - Final product is stable by design (activated carbon), not degradable.

11. **Real-time Analysis:** 2 - Industrial processes enable monitoring but this early report likely limited in advanced real-time pollution prevention tools.

12. **Inherently Safer Chemistry for Accidents:** 2 - High-temperature processing introduces safety hazards.

**B. Circular Chemistry**

1. **Collect & Use Waste:** 5 - Exemplary: upcycling of waste tires into value-added activated carbon.

2. **Maximize Atom Circulation:** 4 - Carbonaceous fraction is reused; low atom economy due to production of volatiles but high carbon circulation.

3. **Optimize Resource Efficiency:** 3 - Converts low-value waste into useful product, with moderate energy input.

4. **Strive for Energy Persistence:** 2 - High-temperature processes typically consume lots of energy; energy recovery possible but depends on the process.

5. **Enhance Process Efficiency:** 3 - Methods can be optimized for industry application; early reports show feasibility.

6. **No Out-of-Plant Toxicity:** 2 - Pyrolysis emissions need management to avoid external toxicity.

7. **Target Optimal Design:** 3 - Product design is targeted (activated carbon with specified porosity).

8. **Assess Sustainability:** 3 - Many studies include process assessments later; original paper may have limited LCA but the concept is sustainable.

9. **Apply Ladder of Circularity:** 5 – Good example for recycling/upcycling (value-added product from waste).

10. **Sell Service, Not Product:** 1 – Historically product-oriented, but activated carbon can be provided as a service (regeneration), not described in the paper.

11. **Reject Lock-in:** 3 - Uses established thermal/chemical infrastructure; adaptable but capital-intensive.

12. **Unify Industry & Policy:** 2 - Industry uptake exists; policy frameworks vary by region.

### S.2.4.2 Waste tires - hard carbon anodes for K/Na-ion batteries [15]

**A. Green Chemistry**

1. **Prevention:** 4 - Upcycling tires into battery anodes prevents landfill and uses waste as feedstock.

2. **Atom Economy:** 3 - Conversion to hard carbon consumes carbon atoms effciently but thermal/chemical treatments produce losses.

3. **Less Hazardous Syntheses:** 2 - High-temperature pyrolysis and chemical activation can involve hazardous steps.

4. **Designing Safer Chemicals:** 3 - Replacing virgin feedstocks with waste reduces upstream impacts; battery materials have their own safety profiles.

5. **Safer Solvents & Auxiliaries:** 2 - Process likely involves thermal and chemical activation rather than benign solvents.

6. **Energy Efficiency:** 2 - High-temperature carbonization is energy-intensive, lowering score.

7. **Use of Renewable Feedstocks:** 5 - Uses waste tires (excellent circular feedstock use).

8. **Reduce Derivatives:** 3 - One-pot conversion approaches can reduce intermediate steps.

9. **Catalysis:** 1 - Process is largely thermal/physical conversion, not catalytic.

10. **Design for Degradation:** 2 - Battery anodes are engineered for stability, not degradation.

11. **Real-time Analysis:** 2 - Process monitoring likely used in pilot settings but not necessarily advanced in publication.
12. **Inherently Safer Chemistry for Accidents:** 2 - Thermal processes and battery material handling require care.

**B. Circular Chemistry**

1. **Collect & Use Waste:** 5 – Example of direct use of waste tires as feedstock for energy storage materials.
2. **Maximize Atom Circulation:** 4 - Carbon is retained and transformed into a high-value application (battery anode).
3. **Optimize Resource Efficiency:** 4 - Replaces virgin hard carbon precursors, improving resource efficiency.
4. **Strive for Energy Persistence:** 2 - High-energy processes limit the energy persistence score unless waste-heat recovery is used.
5. **Enhance Process Efficiency:** 3 - Demonstrated conversion is promising; scale optimization remains to be proven.
6. **No Out-of-Plant Toxicity:** 2 - Process emissions and battery production end-of-life pose potential toxicity concerns.
7. **Target Optimal Design:** 4 - Designing tire-derived carbon for battery performance is a targeted design for reuse in the energy sector.
8. **Assess Sustainability:** 3 - Likely includes performance metrics; LCA is preliminary.
9. **Apply Ladder of Circularity:** 5 - Moves waste up the ladder into high-value component for energy storage, high circularity.
10. **Sell Service, Not Product:** 1 - Potential for battery-as-a-service business models but not addressed in the publication.
11. **Reject Lock-in:** 3 - Using waste feedstock reduces dependency on virgin carbon sources.
12. **Unify Industry & Policy:** 1 - Emerging area; industry/policy integration not yet widespread.

### S.2.4.3 Thermo-oxidative degradation behavior of vulcanized butadiene rubber under thermal recycling conditions [16]

**A. Green Chemistry**

1. **Prevention:** 2 – The study characterizes degradation behavior rather than preventing waste.
2. **Atom Economy:** 2 - Thermal degradation is destructive; atom economy is low.
3. **Less Hazardous Syntheses:** 1 - Thermal degradation can create hazardous volatile organics and emissions.
4. **Designing Safer Chemicals:** 1 - Study focuses on degradation, not safer chemical design.
5. **Safer Solvents & Auxiliaries:** 1 - Not applicable; thermal processes dominate.

6. **Energy Efficiency:** 1 - Thermal recycling is often energy-intensive.
7. **Use of Renewable Feedstocks:** 1 - Focus on waste rubber; while waste feedstock is used, process is destructive.
8. **Reduce Derivatives:** 1 - Decomposition breaks molecules rather than preserving useful derivatives.
9. **Catalysis:** 1 - Thermal oxidative degradation is not catalytic.
10. **Design for Degradation:** 3 - Understanding degradation is a prerequisite for designing materials with better end-of-life properties, but no design principles shown.
11. **Real-time Analysis:** 2 - Materials studies may include in-situ analysis but not necessarily implemented for pollution prevention.
12. **Inherently Safer Chemistry for Accidents:** 1 - Thermal oxidation carries fire/hazard risks.

**B. Circular Chemistry**

1. **Collect & Use Waste:** 3 - Studying the recycling conditions is relevant but the process is destructive rather than upcycling.
2. **Maximize Atom Circulation:** 1 - Thermal breakdown due to formation of volatiles; poor atom circulation.
3. **Optimize Resource Efficiency:** 2 - Provides knowledge to improve recycling efficiency but process remains energy-intensive.
4. **Strive for Energy Persistence:** 1 - Not energy-persistent; high energy input required.
5. **Enhance Process Efficiency:** 2 - Study may inform more efficient thermal recycling but does not present circular end-state.
6. **No Out-of-Plant Toxicity:** 1 - Potential emissions pose out-of-plant toxicity risk.
7. **Target Optimal Design:** 2 - Insights can inform redesign, but the study focuses on analysis rather than on active design optimization.
8. **Assess Sustainability:** 3 - Degradation data can inform future sustainability assessments though a full sustainable solution is not presented.
9. **Apply Ladder of Circularity:** 1 - Thermal degradation corresponds to low-rung circularity (material recovery/destruction rather than reuse/upcycling).
10. **Sell Service, Not Product:** 0 - Not applicable.
11. **Reject Lock-in:** 2 - Understanding degradation may help shift away from incineration/landfill lock-in.
12. **Unify Industry & Policy:** 1 - Technical study alone unlikely to shift policy without broader initiatives.

### S.3.4.4 Behavior of waste tire rubber composites reinforced with waste fibers (2025) [17]

**A. Green Chemistry**

1. **Prevention:** 4 - Turning two waste streams into composites prevents landfill and creates new materials.
2. **Atom Economy:** 3 - Composite formation retains the constituent materials rather than converting them chemically; Atom economy is good.
3. **Less Hazardous Syntheses:** 3 - Mechanical/physical processing is less hazardous than chemical modification.
4. **Designing Safer Chemicals:** 3 - Composites designed for construction or other uses can reduce environmental impacts; safety depends on application.
5. **Safer Solvents & Auxiliaries:** 4 - Processes likely use little/no solvent (mechanical mixing), which is positive.
6. **Energy Efficiency:** 3 - Mechanical processing uses energy but typically less than thermal/chemical conversion.
7. **Use of Renewable Feedstocks:** 4 - Using waste feedstocks is positive for circular feedstock usage.
8. **Reduce Derivatives:** 4 - Direct composite fabrication avoids chemical derivatization steps.
9. **Catalysis:** 0 - Not relevant (no catalysis).
10. **Design for Degradation:** 2 - Composites may be durable but not designed to degrade; end-of-life needs planning.
11. **Real-time Analysis:** 2 - Analysis common in materials testing but not necessarily pollution-preventive monitoring.
12. **Inherently Safer Chemistry for Accidents:** 3 - Mechanical processes are generally safer than chemical ones but still require safety controls.

**B. Circular Chemistry**

1. **Collect & Use Waste:** 5 – Exemplary study: uses waste tires and waste fibers together to make new composite materials.
2. **Maximize Atom Circulation:** 4 - Retains material value in composite form; good atom circulation of solids.
3. **Optimize Resource Efficiency:** 4 - Replaces virgin fillers and matrices, improving resource efficiency.
4. **Strive for Energy Persistence:** 3 - Mechanical recycling has better energy profile than thermal/chemical processes but depends on processing intensity.
5. **Enhance Process Efficiency:** 4 - Straightforward mechanical processing and mixing are efficient and scalable.

6. **No Out-of-Plant Toxicity:** 3 - Solid composites minimize emissions, though any additives must be considered in an out-of-plant toxicity assessment.
7. **Target Optimal Design:** 4 - Designing composites for end-use (construction, automotive parts) is targeted circular design.
8. **Assess Sustainability:** 3 - Likely includes material/property testing; full LCA sometimes absent but but is not precluded by the study design.
9. **Apply Ladder of Circularity:** 5 - Re-use of waste materials into functional products is high on the circular ladder.
10. **Sell Service, Not Product:** 1 - Business model potential exists (e.g., supply-of-materials-as-service) but not described in the article.
11. **Reject Lock-in:** 4 - Uses locally available wastes and common processing equipment — reduces reliance on virgin materials.
12. **Unify Industry & Policy:** 2 - Adoption depends on standards/regulations; paper may suggest applicability but not policy frameworks provided.

# S3 Supporting Info Figure 8

## S3.1 Total Green Chemistry Scores

The total Green Chemistry scores were calculated as the sum of each of the 12 scores, corresponding to each Green Chemistry principle.

| Article / Method | Green Chemistry Total (0–60) |
|---|---|
| 1. [4] (Co-catalyzed MHAT polymer modification, 2024) | 35/60 |
| 2. [8] (Click chemistry polymer functionalization, 2020) | 38/60 |
| 3. [9] (Nitrogen functionalization, ACS talk) | 34/60 |
| 4. [6] (Continuous DMDO polymer epoxidation, 2021) | 40/60 |
| 5. [10] (Hypervalent iodine functionalization, 2020) | 33/60 |
| 6. [11] (Selenium-mediated upcycling of PBD, 2023) | 37/60 |
| 7. [12] (Catechol polymers for water treatment, 2024) | 42/60 |
| 8. [13] (Catalyst-free epoxidation of butyl rubber, 2024) | 44/60 |
| 9. [14] (Activated carbon from waste tires, 2005) | 47/60 |
| 10. [15] (Waste tires - battery hard carbon, 2025) | 48/60 |

| Article / Method | Green Chemistry Total (0–60) |
|---|---|
| 11. [16] (Thermo-oxidative degradation of vulcanized rubber, 2025) | 31/60 |
| 12. [17] (Waste tire rubber composites reinforced with fibers) | 43/60 |

## S3.2 TRL Scale for Chemistry [18]

| TRL | Title (Chemistry/Process) | Description / Key Achievements | Indicators / Criteria (Reaction, Process, Plant, Economics, Work Scale) |
|---|---|---|---|
| **TRL 1** | Idea | First idea of how basic scientific understanding can be turned into a chemical technology; ideation of a new reaction/process. | Identification of target reaction or transformation; rough concept or vision; brainstorming, literature search; no product yet. |
| **TRL 2** | Concept | Formulation of a technology concept or application, initial patent or vision; feasibility of approach is sketched. | Concept formulation, identification of multiple options, patent research, preliminary screening of routes. |
| **TRL 3** | Proof of Concept | Laboratory-level demonstration of the core reaction or functional principle (qualitative or semi-quantitative). | Experimental demonstration of functional reaction, initial kinetics or conversion observed; alternatives evaluated; reaction mechanism hypothesized. |
| **TRL 4** | Preliminary Process Development | Concept validated under lab conditions, scale-up preparation, start of process modeling. | More quantitative reaction studies, property data, simulation models, simple unit operations identified; mass balances; working bench-scale test. |
| **TRL 5** | Detailed Process Development | More rigorous process definition: integration of reaction + unit operations, detailed simulations, bench/demonstration plant models. | Detailed kinetic, thermodynamic, stability data; refined unit operations; scaled mini-plant experiments; integration of upstream/downstream. |
| **TRL 6** | Pilot Trials | Construction and operation of a pilot plant (or pilot-scale units) with partial production, optimization of parameters. | Pilot plant (or pilot modules) operating; optimization of parameters; testing under realistic conditions; long-term stability/durability tests. |

| TRL | Title (Chemistry/Process) | Description / Key Achievements | Indicators / Criteria (Reaction, Process, Plant, Economics, Work Scale) |
|---|---|---|---|
| **TRL 7** | Demonstration / Full-Scale Engineering | Design, construction, and operation of a demonstration plant (or full-scale engineering preparations) with integrated units. | Fully integrated system (reaction + separations) in demonstration environment; specification of full-scale equipment; system performance validated. |
| **TRL 8** | Commissioning | Commissioning and validation of full-scale plant, start-up, fine adjustments, acceptance testing. | Full plant built, tested, audited (site acceptance), performance guarantee, adjustments during start-up, regulatory compliance. |
| **TRL 9** | Production | The technology is commercially operating at full scale over the full operational range, with sustained performance. | The plant is in economic, stable operation; performance meets specifications over time; commercial maturity affirmed. |

### S3.3 Recalculated TRL via the Buchner chemistry-TRL scale [18]

| Article / Method | Revised TRL (chemistry scale) | Justification |
|---|---|---|
| [4] (Co-catalyzed MHAT polymer modification) | **TRL 3** | They have demonstrated the catalytic reaction principle in lab settings, but not yet developed integrated process modules or scale-up. |
| [8] (Thiol-ene click + ROP) | **TRL 3** | Proof-of-concept lab reaction combining click + polymerization investigated; no pilot-level process. |
| [9] (Nitrogen functionalization) | **TRL 3** | Demonstrates reaction modification in lab; hasn’t progressed to integrated process modeling or pilot demonstration. |
| [6] (Continuous DMDO epoxidation) | **TRL 4** | They have validated the reaction in continuous mode, begun process modeling and flow work, moving beyond simple lab demonstration. |
| [10] (Hypervalent iodine functionalization) | **RL 3** | Lab-level reaction validated; no further process development reported. |
| [11] (Selenium-mediated upcycling) | **TRL 3** | Lab-scale upcycling reaction shown; no pilot or process-level integration. |

| Article / Method | Revised TRL (chemistry scale) | Justification |
|---|---|---|
| [12] (Catechol polymer via Friedel–Crafts) | **TRL 4** | Demonstration of one-step functionalization with application (water decontamination) suggests early process modeling beyond pure reaction. |
| [13] (Catalyst-free epoxidation of rubber) | **TRL 4** | They show a practical process at bench scale; method seems scalable and more than pure reaction-level. |
| [14] (Activated carbon from waste tire) | **TRL 7** | This is close to full-scale engineering / demonstration: processes exist in industrial use, conversion from waste to product validated. |
| [15] (Tire → hard carbon anodes) | **TRL 6** | Pilot trials of carbonization and battery testing have been performed; integration toward demonstration scale is ongoing. |
| [16] (Thermo-oxidative degradation behavior) | **TRL 3** | This is largely analysis of degradation behavior (lab-scale), not an integrated process demonstration. |
| [17] (Waste composites with waste fibers) | **TRL 6** | Prototype composite materials have been fabricated and tested; moving toward demonstration-level applications in materials use. |

**References**


1. Januszewski, R., Kownacki, I., Maciejewski, H. & Marciniec, B. Transition metal-catalyzed hydrosilylation of polybutadiene – The effect of substituents at silicon on efficiency of silylfunctionalization process. *Journal of Catalysis* **371**, 27–34 (2019).
2. Orwat, B., Januszewski, R., Dutkiewicz, M. & Kownacki, I. Efficient Transformation of Polybutadienes to Polyolefins: Systematic Studies on the Transition Metal-Catalyzed Hydrogenation of Synthetic Rubbers. *Ind. Eng. Chem. Res.* **62**, 10309–10319 (2023).
3. Shoda, Y., Aoki, D., Tsunoda, K. & Otsuka, H. Polybutadiene rubbers with urethane linkages prepared by a dynamic covalent approach for tire applications. *Polymer* **202**, 122700 (2020).
4. Yin, Y.-N. *et al.* Modifying commodity-relevant unsaturated polymers via Co-catalyzed MHAT. *Chem* **10**, 3088–3099 (2024).
5. Wang, S. *et al.* Modification of polybutadiene with trifluoromethyl and clickable azide groups in one shot. *Polym. Chem.* **12**, 5589–5597 (2021).
6. Ahlqvist, G. P., Burke, E. G., Johnson, J. A. & Jamison, T. F. Continuous dimethyldioxirane generation for polymer epoxidation. *Polym. Chem.* **12**, 489–493 (2021).
7. Xu, L., Jie, S., Bu, Z. & Li, B.-G. Preparation of primary amine-terminated polybutadiene from cis-polybutadiene. *European Polymer Journal* **152**, 110484 (2021).
8. Tian, L., Gu, J., Zhang, H. & Dong, B. Preparation of functionalized poly(1-butene) from 1,2-polybutadiene via sequential thiol-ene click reaction and ring-opening polymerization. *RSC Adv.* **10**, 42799–42803 (2020).
9. Aminomethylation of polybutadiene revisited: Nitrogen-containing block copolymers through post-polymerization modification. https://acs.digitellinc.com/p/s/aminomethylation-of-

polybutadiene-revisited-nitrogen-containing-block-copolymers-through-post-polymerization-modification-35707.
10. Cao, Y., Sayala, K. D., Gamage, P. L., Kumar, R. & Tsarevsky, N. V. Synthesis of Fluorine-Containing Polymers by Functionalization of *cis* -1,4-Polyisoprene with Hypervalent Iodine Compounds. *Macromolecules* **53**, 8020–8031 (2020).
11. Hodges, M. N. *et al.* Upcycling of Polybutadiene Facilitated by Selenium-Mediated Allylic Amination. *Angew Chem Int Ed* **62**, e202303115 (2023).
12. Sehn, T., Kolb, N., Azzawi, A. & Meier, M. A. R. Efficient One-Step Synthesis of Catechol Containing Polymers via Friedel–Crafts Alkylation and Their Use for Water Decontamination. *Macromolecules* **57**, 10802–10811 (2024).
13. Cao, K., Elliott, S., Sirohey, S. A., Durrell, N. & Davidson, G. Fast, Efficient, Catalyst-Free Epoxidation of Butyl Rubber Using Oxone/Acetone for Improved Filler Dispersion. *ACS Omega* **9**, 19601–19612 (2024).
14. Murillo, R. *et al.* Production and Application of Activated Carbons Made from Waste Tire. *Ind. Eng. Chem. Res.* **44**, 7228–7233 (2005).
15. Sun, Q. *et al.* Recycling waste tires as an economical carbon source for developing high-value hard carbon anodes for potassium/sodium-ion batteries. *Sustainable Materials and Technologies* **43**, e01294 (2025).
16. Yue, X., Wan, C., Ren, T., Peng, Z. & Wang, S. Thermo-oxidative degradation behavior of vulcanized butadiene rubber under thermal recycling conditions. *Polymer Degradation and Stability* **232**, 111108 (2025).
17. Haddaji, K., Rim, C. & Boubaker, J. Behavior of waste tire rubber composites reinforced with waste fibers. *Journal of Composite Materials* **59**, 1631–1649 (2025).
18. Buchner, G. A., Stepputat, K. J., Zimmermann, A. W. & Schomäcker, R. Specifying Technology Readiness Levels (TRL) for the Chemical Industry.
